# Effect of forest management choices on carbon sequestration and biodiversity at national scale

**DOI:** 10.1007/s13280-023-01899-0

**Published:** 2023-08-03

**Authors:** Annikki Mäkelä, Francesco Minunno, Heini Kujala, Anna-Kaisa Kosenius, Risto K. Heikkinen, Virpi Junttila, Mikko Peltoniemi, Martin Forsius

**Affiliations:** 1 Institute for Atmospheric and Earth System Research (INAR) & Faculty of Agriculture and Forestry, University of Helsinki, Helsinki, Finland; 2 Finnish Natural History Museum, University of Helsinki, Helsinki, Finland; 3 Department of Economics and Management, University of Helsinki, Helsinki, Finland; 4 Finnish Environment Institute SYKE, Helsinki, Finland; 5 Natural Resources Institute Finland (Luke), Helsinki, Finland

**Keywords:** Carbon sequestration, Forest structure, Harvest level, Photosynthesis, Simulation

## Abstract

Forest management methods and harvest intensities influence wood production, carbon sequestration and biodiversity. We devised different management scenarios by means of stakeholder analysis and incorporated them in the forest growth simulator PREBAS. To analyse impacts of harvest intensity, we used constraints on total harvest: business as usual, low harvest, intensive harvest and no harvest. We carried out simulations on a wall-to-wall grid in Finland until 2050. Our objectives were to (1) test how the management scenarios differed in their projections, (2) analyse the potential wood production, carbon sequestration and biodiversity under the different harvest levels, and (3) compare different options of allocating the scenarios and protected areas. Harvest level was key to carbon stocks and fluxes regardless of management actions and moderate changes in proportion of strictly protected forest. In contrast, biodiversity was more dependent on other management variables than harvesting levels, and relatively independent of carbon stocks and fluxes.

**Supplementary Information:**

The online version contains supplementary material available at 10.1007/s13280-023-01899-0.

## Introduction

Forest covers 70% of the surface area of Finland, with more than 90% of this under forest management and less than 10% strictly protected. Since the middle of the last century, forest management has largely aimed at increasing wood production and thus the harvest potential for forest industries. As a result, we have seen a significant increase in both volume growth and extraction of timber during the last 100 years (Finnish Statistical Yearbook of Forestry 2021). However, increasing concerns about climate change impacts combined with reducing forest carbon stocks and declining carbon sinks, as well as the observed biodiversity loss (Hyvärinen et al. 2019; Kontula and Raunio 2019; Mönkkönen et al. 2022), have put the current methods of forest management under scrutiny. Through decades, forest management has altered the age-structure and functional heterogeneity of the Finnish forests (Gauthier et al. 2015; Korhonen 2021), reducing the number of old-growth forests, large trees and the volume of dead wood in them (Mönkkönen et al. 2022). One solution proposed to stop biodiversity loss has been to increase the coverage and connectivity of protected areas (EU 2020; Duflot et al. 2022). On the other hand, multi-objective forest management has been suggested as a tool to improve the sustainability of forestry under changing climate and increasing wood demand (Díaz-Yáñez et al. 2019; Martynova et al. 2021; Koskela and Karppinen 2021).

In Finland where 60% of forests are privately owned and the average forest estate size of privately owned forests is not more than ca. 30 hectares (Finnish Statistical Yearbook of Forestry 2021), the regulation of forest management has been largely realised through national recommendations developed based on forest science (Äijälä et al. 2019) and administered by forest professionals through a network of consulting companies. Traditionally, the recommendations have been based on economic optimisation of the expected revenue to the forest owner, and they have been developed using empirical growth models of even-aged stands under rotation forestry. More recently, alternative management options have been added to respond to the forest owners’ potential willingness to manage their forests more sustainably. The forest owner may select management chains intended for, e.g., maintaining biodiversity, mitigating climate change or adapting to climate change. However, quantitative research of these alternative management methods is so far rather limited.

Key management options in rotation forestry include rotation length and stocking density, controlled through planting density and thinnings (Niinimäki et al. 2013; Pihlainen et al. 2014; Zubizarreta-Gerendiai et al. 2015). Longer rotations and higher stocking densities have been recommended if the forest owner aims at sequestering carbon into the forest ecosystem, associated with climate change mitigation (Pihlainen et al. 2014). In contrast, recommendations for adaptation to climate change often stress improving forest health and reducing risk of timber loss through increased frequency of natural disturbances, through shorter rotations and lower stocking densities (Möllmann and Möhring 2017). Longer rotations to increase the proportion of old forests have also been recommended for maintaining biodiversity, although it has been recognised that stopping biodiversity loss would require a more holistic view on regional diversity with variable management methods (Duflot et al. 2022) or increasing the area of strictly protected forests (Parviainen et al. 2000; Hanski 2011).

Following the tradition of optimal management guiding the forest owners’ decision making, research comparing alternative management options has usually taken the view of a private forest owner and focused on management units (Eyvindson et al. 2018; Díaz-Yáñez et al. 2019; Duflot et al. 2022). Such studies look at a confined forest area or landscape, devise alternative forest management/set-aside scenarios and analyse the impacts of these on different ecosystem services in the long term. The management scenarios are then evaluated on the benefits they produce, whether economical or some other value-based gains, such as increased carbon sequestration or biodiversity. The implicit assumption in the approach is that the forest owner will always be able to sell all the wood assortments that result from the scenarios, at a given price assumed based on the current market situation. However, there is less research on how the different management alternatives would influence achieving the other related goals, such as carbon sequestration and biodiversity protection, at the national level.

National or multi-national studies on the role of forests in mitigating climate change have been largely related to the level of harvests, determined by the demand of different timber and forest biomass assortments and the potential supply of wood in different regions (Moiseyev et al. 2011; Kallio et al. 2016). Several studies have shown that larger cutting levels function towards reducing the climate change mitigation effect of forests, even if substitution of fossil-based materials with wood is taken into account (Kallio et al. 2016; Kalliokoski et al. 2020; Soimakallio et al. 2021). Because cutting levels can be thought to be related to the mean rotation length, longer rotation increasing and shorter decreasing the average growing stock (Heaps 2015), these national level results would seem to be in line with the management recommendations described above. However, while management strategies may be crucial for determining the level of cuttings at the forest estate level, at the national level cuttings are also strongly constrained by the demand of wood, as demonstrated using global market partial equilibrium models (Moiseyev et al. 2011; Kallio et al. 2016). The demand levels are reflected in timber prices, which is one possible way of including the impact of demand on estate-level optimisation (Eyvindson et al. 2021). However, the interplay of management recommendations and cutting levels/wood demand at the national level therefore still largely remains to be investigated.

In modelling studies, the effect of management on forest biodiversity has been assessed through various indicators that are derivable from the variables predicted by the forest model (e.g., Thom et al. 2017). The indicators may include direct forest variables such as tree species diversity, but more general biodiversity indicators are those that either reflect forest stands with high species richness or specifically the suitability of the forest as habitat for red-listed mammal, avian, invertebrate or fungal species (Edenius and Mikusinski 2006; Thom et al. 2017). A straightforward biodiversity indicator included in many model studies (e.g. Akujärvi et al. 2021) is the amount of coarse deadwood which is a critical resource in boreal forests for ca. 4000–5000 species (20–25% of all forest-dwelling species), including many rare and red-listed insects or fungi (e.g. Siitonen 2001; Tikkanen et al. 2007). A complementary approach is to directly estimate the presence of a set of species by using empirical and expert knowledge of species ecological habitat requirements and models that link species presence to habitat features (Edenius and Mikusinski 2006; Tikkanen et al. 2007). The value for modelling species instead of structural or stand quality-based biodiversity indicators, such as tree diversity and amount of dead wood, is that species models can account for interactions of multiple habitat features and therefore provide a more realistic biodiversity responce to management actions that alter the variables determining habitat suitability. A number of recent studies have developed and applied such habitat suitability index (HSI) approach for Fennoscandia for a variety of avian and mammal species that together provide a broad representation of different factors affecting biodiversity (Mönkkönen et al. 2014, 2022; Duflot et al. 2022).

In addition to the potential effect of alternative management recommendations, we also need to know how many forest owners would be willing to adopt the new recommended methods. According to a Finnish forest-owner study by Koskela and Karppinen (2021), only less than 10% of forest owners would be willing to implement voluntary permanent protection in their forests, whereas ca. 20% would be prepared to do the same if it was compensated. Similarly, 20% of forest owners were classified as “promoters of biodiversity through forest management”, while an additional 16% were seen as “moderate conservationists” who might be prepared to undertake some of the conservation or management measures but without an equally clear determination. Is this level of willingness sufficient to increase the biodiversity and carbon related values of the forests? How would the measures affect the potential harvest, if implemented? Would it be better to protect a larger part of all forests and manage the rest more intensively, or would it be more effective to apply multipurpose forestry more extensively everywhere? If a “best solution” was found, how would the associated forest management practices be received by forest owners?

The objective of this study is to compare the contribution of harvest level, management strategy and percentage of strictly protected area for carbon sequestration and biodiversity potential in forests. We consider the harvest level either as a direct result of management strategies through rotation length and thinning intensity, or as a fixed total harvest determined at country level by roundwood demand. We will define alternative management strategies through different management actions, such as species selection, rotation length, and thinning intensity, taken at the management unit level. The main focus of the protected area analysis will be on the potential of extended protected area to safeguard carbon sequestration and biodiversity when the harvests are kept at a fixed level. We do the analysis for Finland, by means of model simulations that focus on the impacts of different management strategies on forest growth, carbon stocks and fluxes as well as selected biodiversity indicators.

To achieve the above-stated research objectives, we aim to answer the following specific research questions (RQ):

1. If total harvest is unconstrained, how do different management strategies affect harvest levels, wood production, carbon sequestration and biodiversity indicators?
2. If total harvest is constrained, how do different management strategies affect wood production, carbon sequestration and biodiversity indicators?
3. In a comparison of multi-objective forest management vs increased set-aside for protection, which of these could better achieve national goals of increased carbon sequestration and biodiversity protection?

## Materials and methods

### The C balance model

The forest scenarios were simulated with the forest growth and carbon balance model PREBAS, which combines a forest growth model (Valentine and Mäkelä 2005), and a forest gas flux model (Peltoniemi et al. 2015). The tree growth model is cohort-based and can be applied to different stand structures (Hu et al. 2023) but is here used as a stand mean-tree model by species, including *Pinus sylvestris*, *Picea abies* and *Betula* spp. The model derives growth from carbon acquisition and allocation at annual time resolution. Mean trees are described in terms of 13 variables, including component biomasses and crown, stem, and root system dimensions. Growth is assumed to follow from net annual photosynthesis, allocated to the different biomass components to maintain structural rules. Species interactions are described through effects on light availability for photosynthesis. The gas flux model is an ecosystem model of intermediate complexity (Peltoniemi et al. 2015). The model works at a daily time-step interlinking gross primary production (GPP), evapotranspiration, and soil water. The gas flux model has been calibrated using GPP and water balance data from 10 eddy covariance sites in Fennoscandia (Minunno et al. 2016) and the whole PREBAS model has been calibrated using growth experiments in Finland (Minunno et al. 2019). After calibration, the mortality rate in unmanaged forests has been modified in PREBAS by including an additional mortality term based on an extensive empirical study in Fennoscandia (Siipilehto et al. 2020; Supplementary Information 1). The mortality of trees larger than 10 cm is used as input to the coarse deadwood module which decomposes the dead trees using the decay model by Mäkinen et al. (2006). So as to complete the forest ecosystem carbon cycle, a ground vegetation module has been added to PREBAS based on empirical results from ground vegetation inventories (Tonteri et al. 2005; Muukkonen et al. 2006; Lehtonen et al. 2016), (Supplementary Information 2).

For stands growing on upland soils, PREBAS is linked with the soil carbon balance model Yasso, allowing us to estimate the whole ecosystem carbon fluxes and storages (Tuomi et al. 2009, 2011). For stands in drained peatland soils, soil respiration estimates were based on measurements representing both peat decomposition and litter decomposition (Ojanen et al. 2010, 2013; Minkkinen et al. 2018).

### Biodiversity indicators

Biodiversity was analysed with previously published habitat suitability indices (HSI) for selected species (Mönkkönen et al. 2014) (Supplementary Information 3), together with deadwood volume and birch volume. The HSI included five bird species (capercaillie (*Tetrao urogallus*), hazel grouse (*Bonasa bonasia*), three-toed woodpecker (*Picoides tridactylus*), lesser-spotted woodpecker (*Dendrocopos minor*), long-tailed tit (*Aegithalos caudatus*)), and one mammal species [Siberian flying squirrel (*Pteromys volans*)]. The values of HSI index vary between 0 and 1 indicating the relative habitat suitability for species based on forest attributres. For each species separately and for each simulation run, we derived the HSI values by applying the species-specific HSI formula to the stand structural variables produced by PREBAS, such as tree species absolute and relative volume, basal area, density, age, and basal area of recently died trees. The six indicator species have partly divergent ecological preferences but together they reflect various complementary aspects of biodiversity values in boreal forests. In addition to the indices, we also employ the volume of deadwood and deciduous volume (represented by *Betula* spp. in PREBAS) as biodiversity-related indicator variables (Felton et al. 2022).

### Input data, simulations and presentation of results

PREBAS initialization requires basic stand-mean variables by tree species (mean height and breast height diameter, basal area) in addition to site fertility class (quantified as site type, Cajander 1949). These were obtained from publicly available, spatially explicit National Multisource Forest Inventory data (MSNFI) (Mäkisara et al. 2019). These data are available in a 16 × 16 m grid which has been further elaborated into segments combining adjacent pixels representing similar stand characteristics. The segments form unified units or “stands” of variable size (mean 3926 m^2^) (Haakana et al. 2022). In addition, we used information about the field-based NFI on region-specific age-class distributions to correct the MSNFI-based distribution which has been found to be slightly skewed (Haakana et al. 2022) (Fig. S4.1, Supplementary Information 4). The region-specific age distributions were represented by the areas of forests in these classes, and they were calculated by 20-year interval for the forests in forest land. Forest land contains the productive forest land used for wood production.

The climatic inputs to the gas exchange module include daily mean temperature, vapour pressure deficit, precipitation and photosynthetic photon flux density. These were available as scenarios on a national grid at 10 × 10 km resolution from the Finnish Meteorological Institute and were constructed by interpolation based on measured data from the national meteorological station network during the period 1971–2020. For future simulations, years were sampled randomly from this data period.

The Yasso model requires a spin-up for initialisation of the soil carbon storage components (Liski et al. 2005). This involves running the model to steady state with litter fall and weather inputs representing the standard management and current weather (repeating weather data from 1971 to 2020). Yasso initialisation was carried out for each sampled segment.

**Fig. 1:**
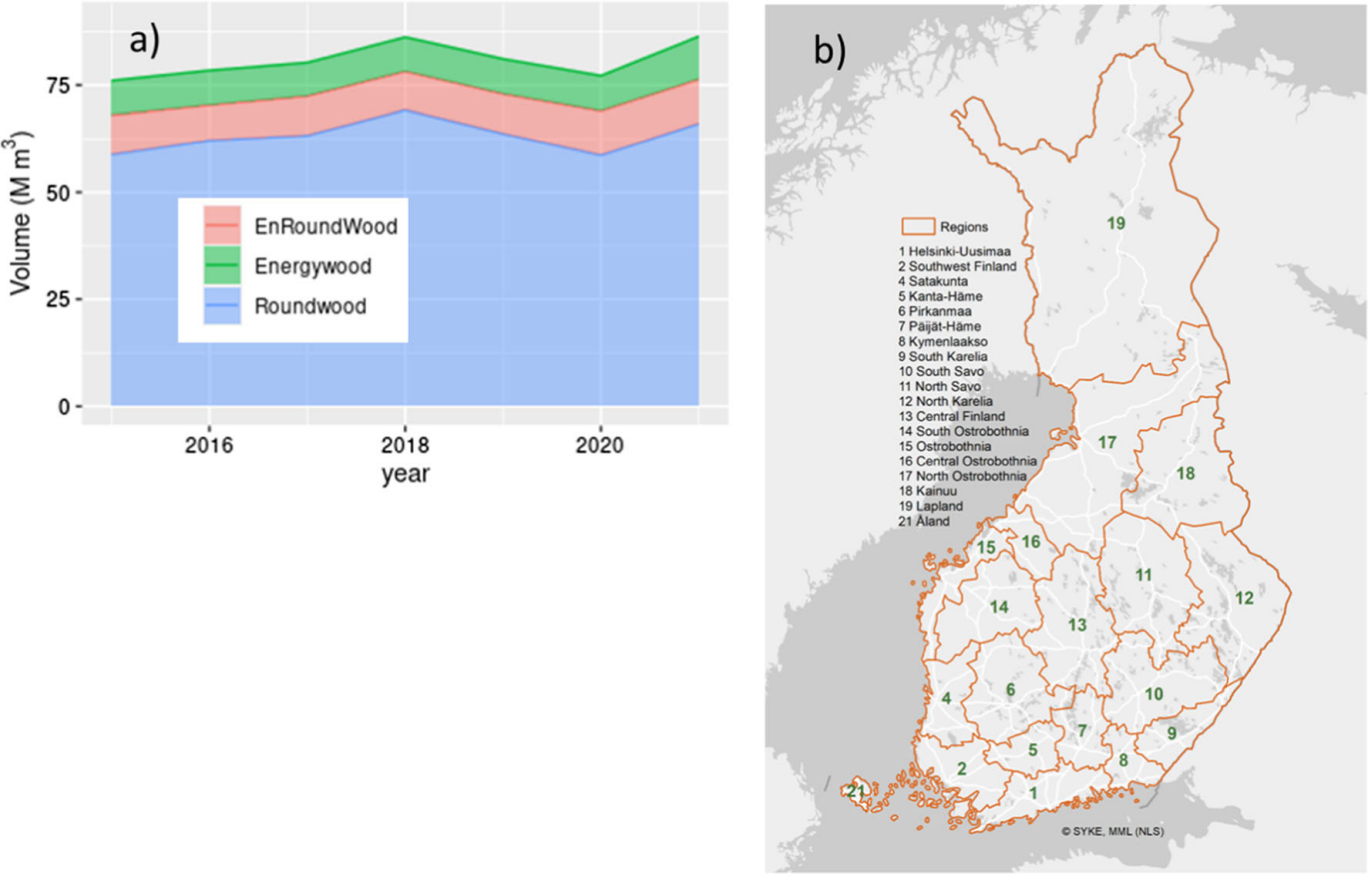
**a** Harvests during 2015–2020, split to round wood, energy wood from round wood and energy wood from harvest residues. Round wood consists of stems and is further divided into parts used for energy (EnRoundWood) or timber (pulp and sawn timber) (Roundwood). Energy wood (Energywood) includes harvest residues consisting of stumps, branches and stem parts of low dimensions (Finnish Statistical Yearbook of Forestry 2021). **b** Administrative regions in Finland

In addition, model inputs included management that consisted of management actions specific to each management strategy, and a predetermined or free cutting level (see Sect. "Management strategies and cutting levels"). These were defined relative to a Base management strategy and a historical Business As Usual (BAU) cutting level based on national statistics for the initial simulation period 2015–2020 (Fig. 1a).

The simulations were carried out for 2015–2050. This period was chosen because we wanted to link the results with projected industrial emissions that were available up to 2050 (Forsius et al. 2023). The simulations were conducted by administrative region. For each of the 19 regions (Fig. 1b), we sampled 20 000 segments for the simulation, and these were followed for the length of the run. The sampling was stratified based on species-specific age classes to correct for the biased age-class distribution in the MSNFI data. If the sample came from the protected area no management was done. In managed forest, we checked each year if either clear cut or thinning conditions were met, and if so, these operations were performed, until the harvest limits for the region were reached. This means that harvest allocation was random among stands mature for thinning or clearcut, respectively. In addition, to provide a realistic split between clear cuts and thinnings, we used a statistics-based annual maximum clear-cut area. If this was reached before the harvest limit, the rest of the harvests were conducted as thinning. Energy wood from harvest residues and stumps was similarly collected until the respective prescribed limits were reached.

In the results, we present all variables as national means standardized with respect to total forest area (forest land classes 1 and 2), including protected areas, or separately for managed and protected forest. For the C balance, we used six PREBAS outputs as indicators of national carbon balance (Table 1). Total volume is of interest for timber production, while total ecosystem carbon reflects the carbon storage capacity of forests. Gross volume increment is a proxy for forest productivity, which depends not only on climatic and edaphic factors, but also on stand structure and age distribution. The latter is summarised in mean forest age; decreasing mean age is indicative of decreasing harvest potential. Net ecosystem production measures the instantaneous sink strength of the forest, while net biome production accounts for the effect of harvests on the net sink.

**Table 1:** Model outputs used as indicators of national carbon balance and biodiversity. Unless otherwise stated, all values are national means standardized with respect to total forest area, including protected areas. *CB* carbon balance, *BD* biodiversity, *GPP* gross primary production, *RE* total ecosystem respiration, ∆*C*_TOT_ net change in total ecosystem carbon, *H* harvest removals, *HIS* habitat suitability index

Role	Variable	Name	Meaning	Unit
CB	Stem volume	*V*	Current standing volume	m^3^ ha^−1^
	Total ecosystem carbon	*C*_TOT_	C in trees, soil and ground vegetation	Mg C ha^−1^
	Gross volume increment	*G*_V_	Gross volume growth before removals and mortality	m^3^ ha^−1^ year^−1^
	Mean stand age	*a*	Age of dominant layer	year
	Net ecosystem production	NEP	GPP - RE	g C m^−2^ year^−1^
	Net biome production	NBP	∆*C*_TOT_ = NEP - H	g C m^−2^ year^−1^
BD	Capecaillie	HSI1	suitability index with values in [0,1]	–
	Hazel grouse	HSI2	- “ -	–
	Three-toed woodpecker	HSI3	- “ -	–
	Lesser spotted woodpecker	HSI4	- “ -	–
	Long-tailed tit	HSI5	- “ -	–
	Siberian flying squirrel	HSI6	- “ -	–
	Deadwood volume	*V*_DW_	Decaying volume of large dead trees	m^3^ ha^−1^
	Deciduous volume	*V*_DEC_	Volume of *Betula* spp.	m^3^ ha^−1^

Following Duflot et al. (2022), we used a threshold HSI to indicate habitat suitability. Therefore, for the HSI means, only values > 0.5 were accounted for (the rest were set to 0), with the assumption that sites with HSI < 0.5 are not suitable. In addition to temporal development graphs, we also calculated temporal scenario means over the period when the different scenarios were applied, i.e., 2021–2050.

### Management strategies and cutting levels

Forest management strategies were described based on (1) national recommendations (Äijälä et al. 2019) and (2) modifications to these as defined by a stakeholder meeting organized by the IBC-CARBON project^1^ (Supplementary Information 4). The national recommendations were those prescribed for rotation forestry aiming at maximized revenue from roundwood production, complemented with recommendations for energywood harvests. The reference recommendations include choice of species and stocking density at establishment, timings and intensities of thinnings and rotation length. Both thinnings and clearcuts are based on stand structure using harvest models prescribed in the recommendations (Äijälä et al. 2019). Harvests are carried out when the stand reaches a certain dominant-height-dependent basal area which depends on species, site type and effective temperature sum. The intensity of harvest also depends on stand characteristics, and the harvest models allow for some variability in both timing and intensity. Clear cuts, on the other hand, are triggered by stand mean diameter or age by species, site type and effective temperature sum (Äijälä et al. 2019).

The alternative management strategies were defined as modifications to the reference strategy (termed Base strategy from here onwards) in relation to the three objectives, (1) climate change adaptation, (2) climate change mitigation through forest C sequestration, and (3) biodiversity protection. The modifications were formulated based on a full-day stakeholder workshop where researchers and forestry professionals shared their ideas and expertise on impacts of forest management measures. The alternative measures consisted of changed rotation lengths and thinning intensities, choice of species at establishment and favouring certain species at thinnings. In addition, the adaptation strategy included fertilization at selected sites, where fertilization was implemented as changing the growth site to the next more productive type for the next 10 years, then reducing back to the original in the following 10 years. The protection strategy included leaving an unmanaged buffer zone of 200 m around protected areas, as well as leaving sufficiently large (> 20 cm) retention trees at final harvest. The results of the stakeholder meeting were interpreted in terms of the modelled forest management actions and implemented in PREBAS as alternative forest management strategies (Table 2).

**Table 2:** Definition of alternative management strategies, defined relative to Base strategy (see text; Äijälä et al. 2019)

Measure	Climate change adaptation	Climate change mitigation	Biodiversity protection
Regeneration	Species to fit the site	Same species as before	Same species as before
Species	Favour broad-leaf mixtures (+ 20% birch at plantation)	No favouring of species	Birch mixture with spruce and pine at least 20% of stocking
Rotation	Shortened by 5–10 years	Lengthened by 25%	Lengthened by 25–30%
Thinnings and tending	On time	High thinning at age > 50	None specified
Harvest residues	Collected as in base strategy	Leave on site	Leave on site
Fertilisation	At sites poorer than mesic heath	No	No
Retention trees	No	No	Leave trees larger than 20 cm as retention trees (incl. broadleaves), 5–10% of harvest volume in total
Cutting rules if supply greater than demand	None specified	Cuttings from the most productive sites preferred (site classes 1 and 2) No cuttings from forests older than 120 year	Cuttings from the most productive sites preferred (site classes 1 and 2) No cuttings from forests older than 120 year
Protection areas	No	No	Buffer zones of 200 m around protected areas

For the constrained total cutting levels (RQ2 and RQ3) we applied three different cutting levels (Huttunen et al. 2022): Business as Usual (BAU, ~ 80 Mm^3^ year^−1^) was based on realized cuttings during 2015–2020 (Fig. 1a), High Harvest level amounted to ~ 1.2 × BAU, and the Low Harvest level was approximately 60% of BAU. Forthly, we ran a no-harvest simulation (NoHarv). The harvest levels were implemented by administrative region (Fig. 1b) on the basis of the realized shares of the regions in the recent history.

### Scenarios

Three types of management scenarios were devised to answer our three research questions. The scenario types incorporated management strategies, cutting levels and types of set-aside in different combinations. They were termed (1) Management-driven, (2) Demand-driven, and (3) Forest owners’ preference. All scenario types were applied to either current or extended protected area. In all scenarios, the management was applied in productive forestry land (forest land classes 1 and 2 in forestry statistics), while protected areas and unproductive forest land (land class 3) were left unmanaged. The definitions of the scenarios generated a variable number of simulation runs each (Table 3).

**Table 3:** Simulated management and harvest scenarios. See text for details. Pure strategy = one management strategy (Base, Adaptation, Mitigation, Protection) applied to 100% of stands throughout the country. Current = current protection areas, totaling 8.5% of national forest area. Extended = current + extended protected areas, the total reaching at least 10% of total area in each region. *RQ* research question

Scenario type	Protected areas (RQ3)	Management	Cutting levels	# runs
Management-driven (RQ1)	Current	All pure strategies	Not constrained	4
	Extended	All pure strategies	Not constrained	4
Demand-driven (RQ2)	Current	All pure strategies	All cutting levels incl. NoHarv	13
			BAU obtained from regional mix	4
	Extended	All pure strategies	All cutting levels	12
Forest owners’ preference (RQ3)	Current	0.8 Base + 0.2 Mitigation	BAU	1
	Current + additional 5.2% set-aside	0.8 Base + 0.148 Mitigation	BAU obtained from 0.948 original forest area, 0.052 NoHarv	1

In all scenario types, we varied the set-aside between the current strictly protected area (8.5% of total simulated forest area) and additional set-aside to guarantee at least 10% in all administrative regions. Because the share of protected areas is > 10% in northern Finland, this led to 13.7% protected area in total. The extended protected area was selected on the basis of its biodiversity values, as explained in Forsius et al. (2023). In the Protection management strategy that included the 200-m buffer zones around the original protected areas, the total protected area proportion was 11.5%.

Under the “Management-driven” scenarios we varied the management strategy (Base, Adaptation, Mitigation and Protection) and always managed all stands according to strategy rules, without any upper or lower harvest limit.

The “Demand-driven” scenario type covered the combinations of the four management strategies (Base, Adaptation, Mitigation and Protection) with each cutting level (BAU, High and Low, NoHarv). In addition, we simulated all demand-driven management strategies with a scenario that followed the BAU cutting level nationally, but where the regional cutting levels were varied between regional BAU, High and Low levels. The Low level was assigned to the regions that had relatively very high harvest levels in the historical data (Kanta-Häme, Päijät-Häme, Kymenlaakso, South Carelia, South Savo), and the consequently reduced cut was compensated by assigning the High cutting level to regions where past relative cuttings were lower (Pirkanmaa, North Savo, North Carelia, Central Finland, North Ostrobothnia) (Junttila et al. 2023).

Finally, the “Forest owners’ preference” scenarios were devised as demand-driven (BAU) simulations where management strategies were mixed based on information about forest owners’ stated willingness to adopt alternative management strategies or set-aside. According to a forest owner survey (*N* = 405) administered in the beginning of 2019 using the consumer panel of the professional polling company, approximately 20% of forest owners, independently of the size of the holding, were willing to undertake management actions similar to our mitigation strategy (Table 2) (Salenius and Kosenius, unpubl.). In addition, assessed based on the random-sampling forest owner survey data (*N* = 5010) administered in 2020, and taking the non-response rate into account, less than 5% of forest owners would be prepared to set aside forest land for protection (Salenius and Kosenius, unpubl.). Drawing from this information, we simulated two mixed strategies under BAU harvest level, M20: 20% mitigation combined with 80% Base, and MSA20: a total of 20% mitigation and set aside combined with 80% Base, where we used the additional set aside proportion from the extended protection scenarios. This varied by region, averaging at 5.2% for the whole country (8.5% current protected area + 5.2% additional set aside).

The management-driven scenarios informed our RQ1 and the demand-driven scenarios RQ2. In all scenario types, the comparison between current and extended protected areas served to answer RQ3. In addition, the forest owners’ preference scenarios were used to shed light on RQ3, as they provided a comparison of the effectiveness of modifying management strategies versus increasing the area of protected forest, both constrained by the same fixed harvest level. This simple example of alternative combined scenarios is of interest because it seems feasible in the light of the owner survey.

## Results

### Management-driven simulations (RQ1)

**Fig. 2:**
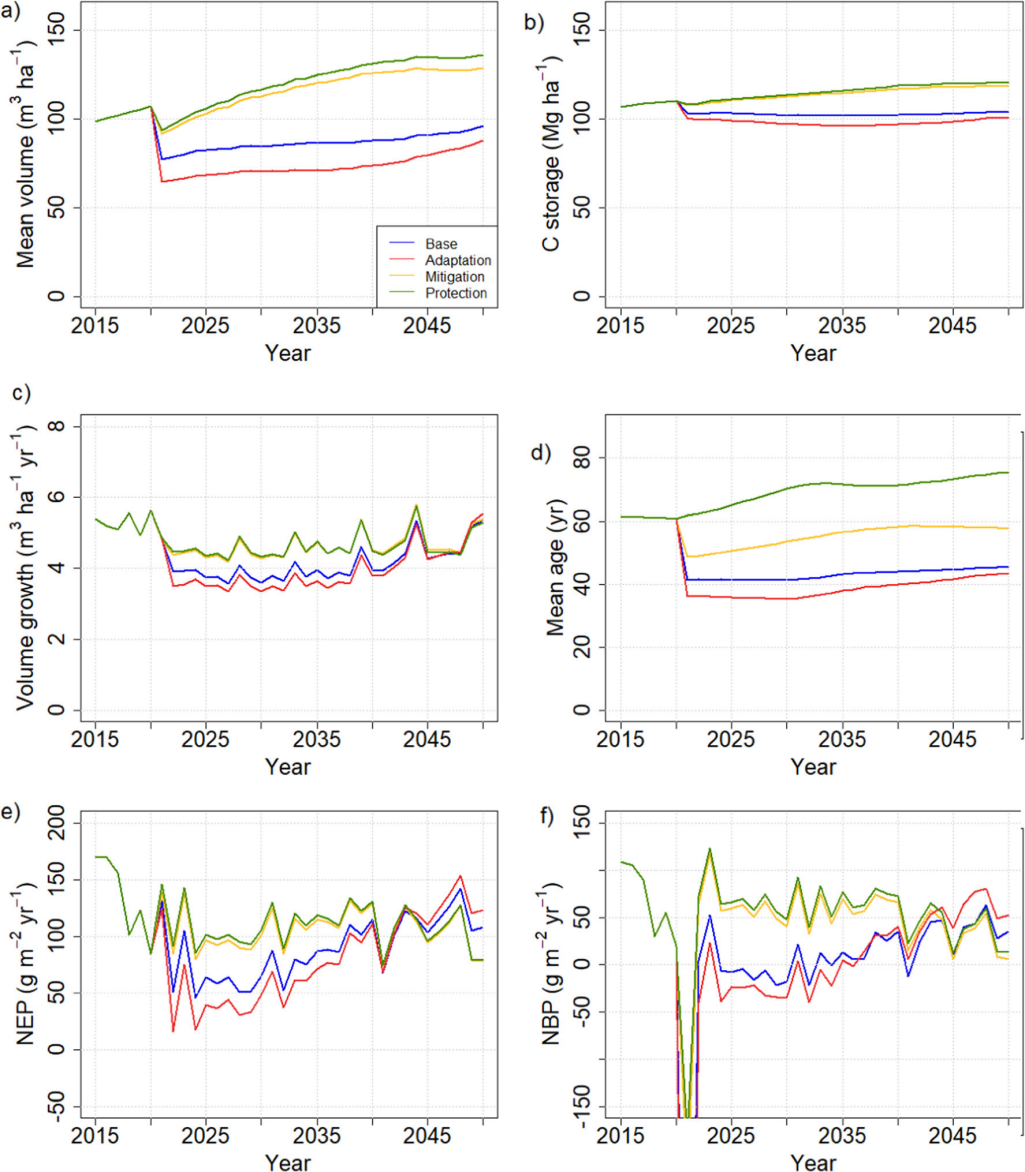
Comparison of management strategies in management-driven scenarios. Results are shown as country means per unit area. **a** Standing volume, **b** total C stock in trees, soil and ground vegetation, **c** annual growth, **d** mean stand age, **e** net ecosystem production (NEP), **f** net biome production (NBP) = net ecosystem production—harvests

**Fig. 3:**
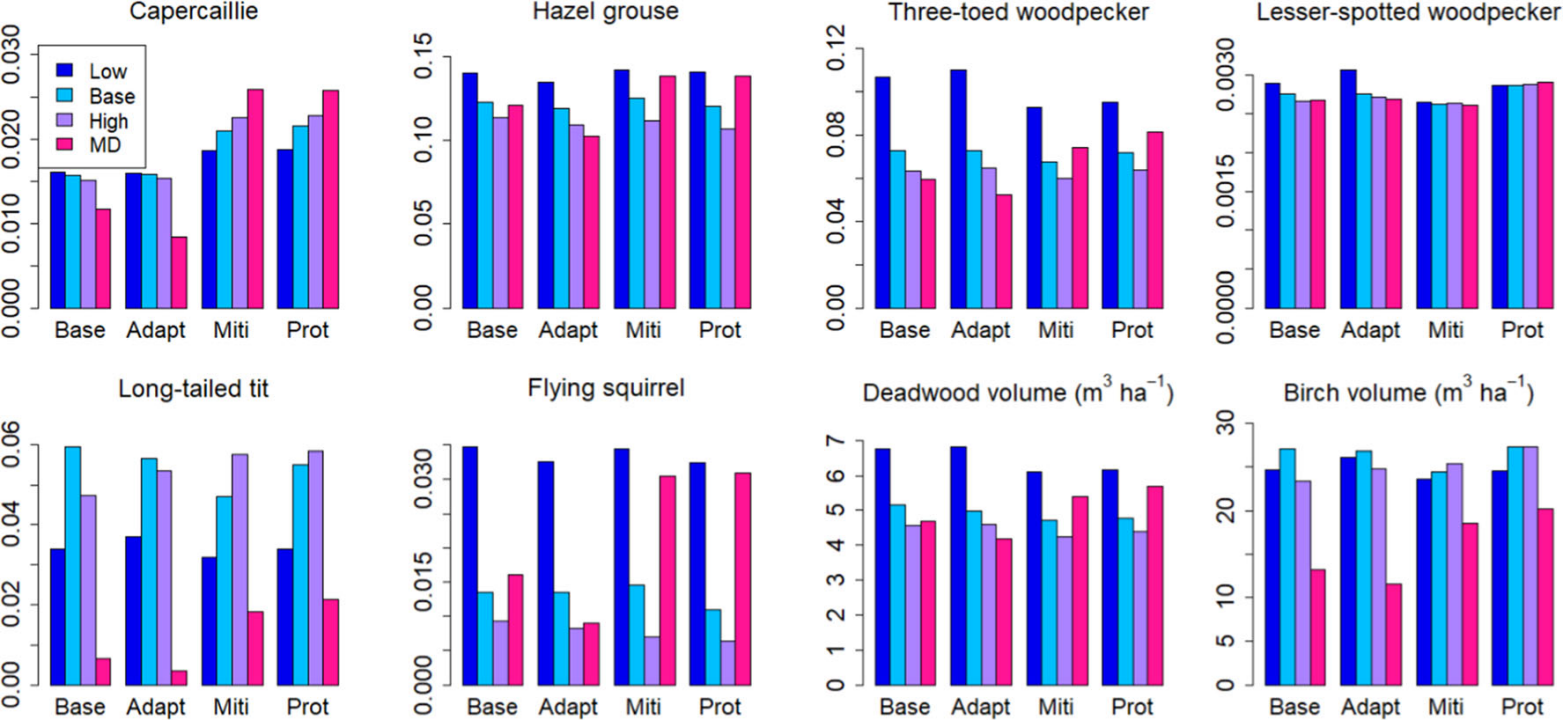
Temporal mean values over 2021–2050 of biodiversity indicators (spatial means of values > 0.5, else taken to be 0) under different management strategies with current protection areas. Low, Base and High indicate harvest levels in demand driven scenarios. *MD* management driven scenarios

The management-driven simulations with current protection areas led to clear differences between management strategies in both carbon-balance related variables (Fig. 2) and biodiversity indicators (Fig. 3). The mitigation and protection strategies led to higher standing volumes, total carbon stocks as well as higher carbon sequestration than the base or the adaptation strategy, the latter showing consistently the lowest values of stocks and fluxes. The biodiversity indicators, shown as temporal means, attained higher values for the protection and mitigation strategies for all but one indicator, the lesser-spotted woodpecker. In all other cases, the mean values for mitigation and protection were the highest and for adaptation the lowest, although a lot of both temporal and spatial variability was embedded in the indicators (not shown).

**Fig. 4:**
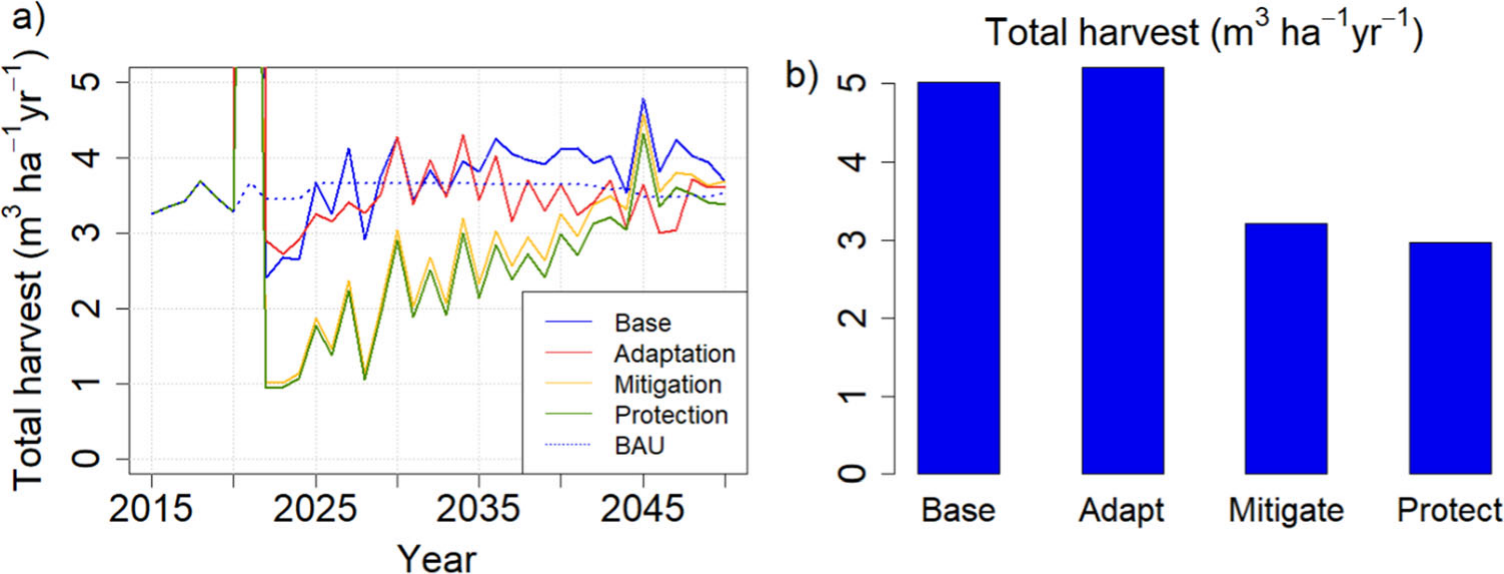
Per-hectare cutting levels under different management-driven scenarios from a total area of 23.4 Mha. **a** Temporal development compared with BAU harvest level. **b** Mean per-hectare annual cut during the simulation period with divergent management (2021–2050) with current protected areas

With no cutting limitations, the scenarios created their own cutting levels (Fig. 4a). These turned out to be very volatile, including an initial harvest in the first year of application that ranged from 10 (protection) to 60 m^3^ ha^−1^ year^−1^ (adaptation), then reducing to levels considerably lower than current annual cut (ca. 3.5 m^3^ ha^−1^ year^−1^), with an upward trend towards the end of the simulation. As temporal means during the application period of the alternative strategies (2021–2050), the mitigation and protection strategies led to the lowest harvest levels, representing about 60% of the base and adaptation harvest levels which resembled each other (Fig. 4b). Cutting levels of all scenarios approached each other over time (Fig. 4a).

### Demand-driven simulations (RQ2)

**Fig. 5:**
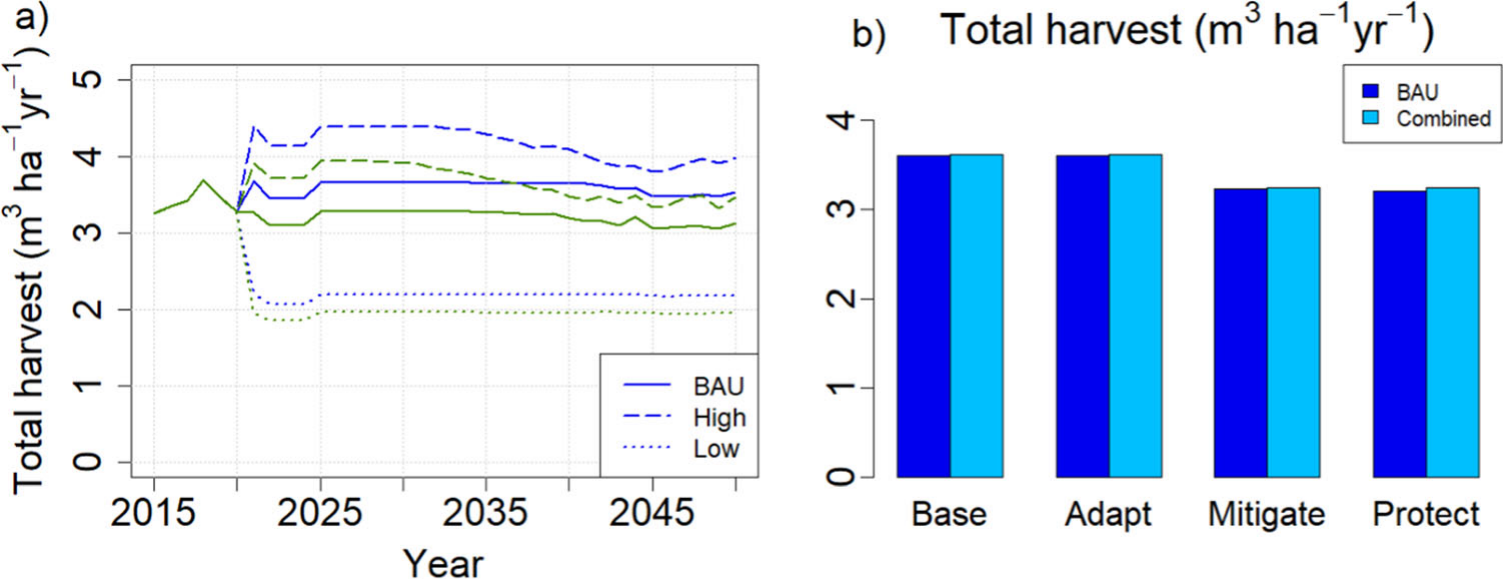
**a** Development of mean per-hectare harvest levels from a total area of 23.4 Mha. Blue lines: Base and Adapt (roundwood and harvest residues); green lines: Mitigate and Protect (roundwood only). Harvest levels in Base and Adapt strategies are larger than those in Mitigate and Protect strategies because the latter do not include energy wood from harvest residues (Table 2, Fig. 1a). **b** Temporal mean harvest levels during 2021–2050. Combined = BAU harvest level reached nationally by mixing High, Low and BAU in selected regions (see text, Table 3)

In the demand-driven simulations, the set harvest levels are followed as long as there are forest stands where the management strategies allow for harvests, either clear cuts or thinnings. The set levels were always reached with the Low harvest level, but could not be reached throughout the simulation period with the BAU and High harvest levels. This failure was caused by those few regions where the historical reference harvest levels had been high, and could be corrected by applying the mixed BAU scenario that combined regional High, Low, and BAU harvest levels. However, the implied difference in harvest levels between the original and combined BAU at the country level was negligible (Fig. 5).

**Fig. 6:**
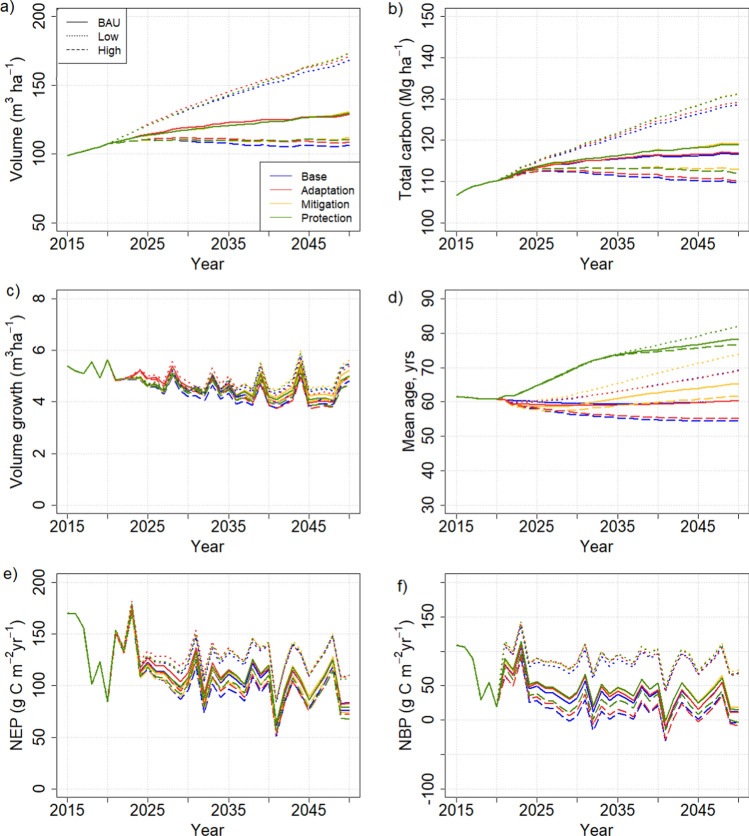
C balance indicator variables in demand-driven scenarios with current protection area. **a** Standing volume, **b** total C stock in trees, soil and ground vegetation, **c** annual growth, **d** mean stand age, **e** net ecosystem production (NEP), **f** net biome production (NBP) = net ecosystem production—harvests

Contrary to the management-driven scenarios, any management strategy effects were dominated by impacts of the harvest level in the demand-driven scenarios. This was especially the case for the C balance indicators (Fig. 6), whereas the BD indicators were affected by management as well (Fig. 3). Five of the eight BD indicators showed a clear dependence on cutting level, four attaining higher (hazel grouse, three-toed woodpecker, flying squirrel, deadwood volume) and one (long-tailed tit) lower values with lower cuttings, while one was more affected by management strategy (capercaillie). The remaining two were largely unaffected by either management strategy or harvest level (Fig. 3). The national averages were insensitive to the regional distribution of harvest levels in the “BAU obtained from regional mix” scenario (Table 3) (not shown).

### Set-aside vs multi-objective management (RQ3)

**Fig. 7:**
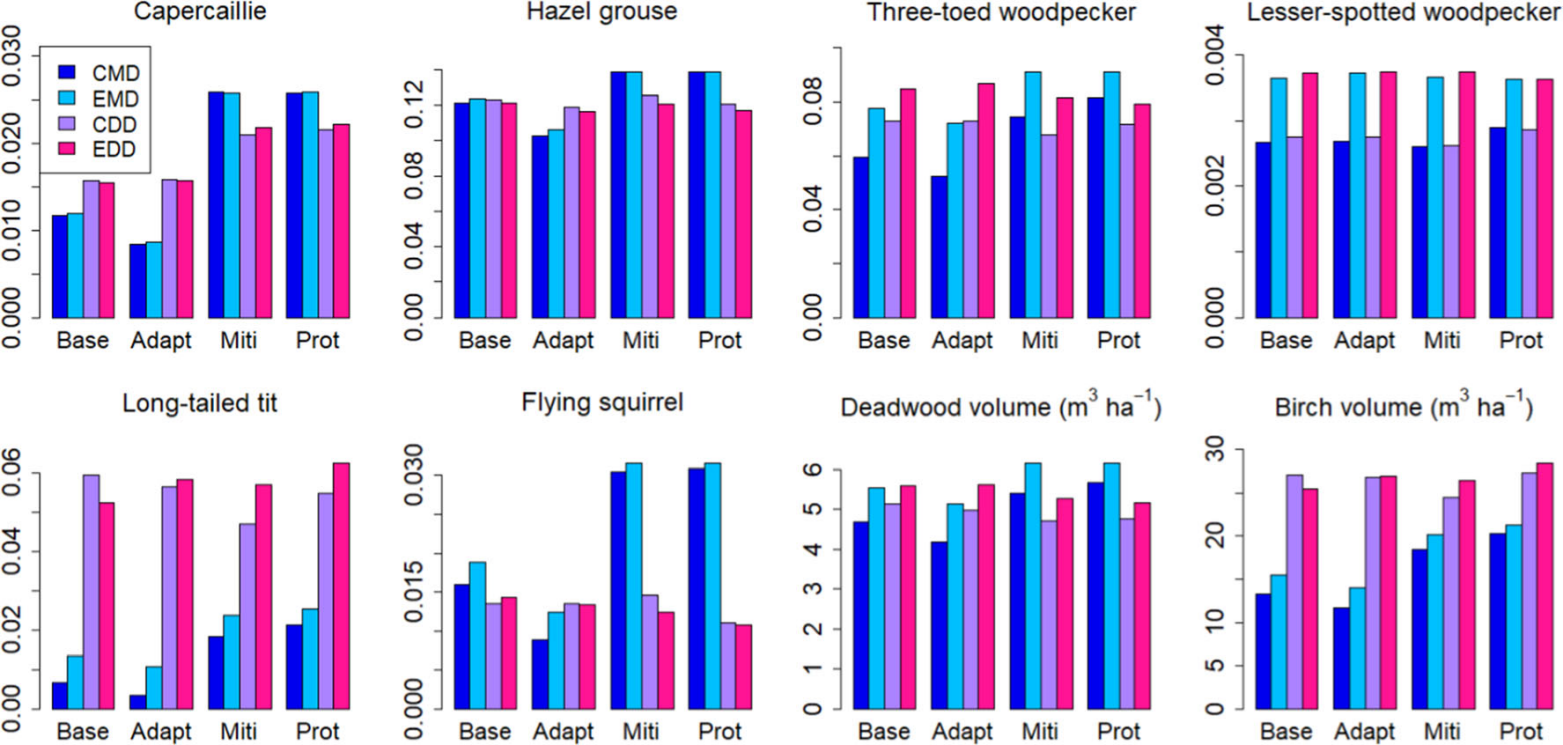
Temporal mean values over 2021–2050 of biodiversity indicators (spatial means of values > 0.5, else taken to be 0) under different management strategies. *CMD* management-driven scenarios with current protection areas, *EMD* mangement-driven scenarios with extended protection areas (at least 10% set aside by all regions), *CDD* demand-driven scenarios with BAU harvest level and current protection areas, *EDD* demand-driven scenarios with BAU harvest level and extended protection areas

Extending the protected area in the management-driven scenarios implied that both C balance and BD indicators reached higher values than with the original protection areas (Figs. 7, S6.1a). Total cutting levels reduced slightly under the extended protection scenarios but the difference between current and extended protection was less than that between management strategies (Fig. S6.1b, supplementary Information).

In the demand-driven scenarios, where the national harvest levels were maintained regardless of the size of the protected area, the per-hectare harvests in managed forest consequently increased (Fig. S6.2, supplementary Information). In the areas with high historical reference cuttings this led to earlier failure to reach the target than under current protected areas, and hence yielded somewhat lower realized time-average cuttings (Fig. 5). However, the C balance indicators at national level were not affected by this change, except that NBP increased slightly in response to lower realized cutting levels in the BAU and High scenarios (Fig. S6.2, Supplementary Information).

Three biodiversity indicators (three-toed woodpecker, lesser-spotted woodpecker and deadwood volume) increased in the BAU harvest level under the extended scenario (Fig. 7), and a fourth (Siberian flying squirrel) under the High harvest level (Fig. S6.3, Supplementary Information).

**Fig. 8:**
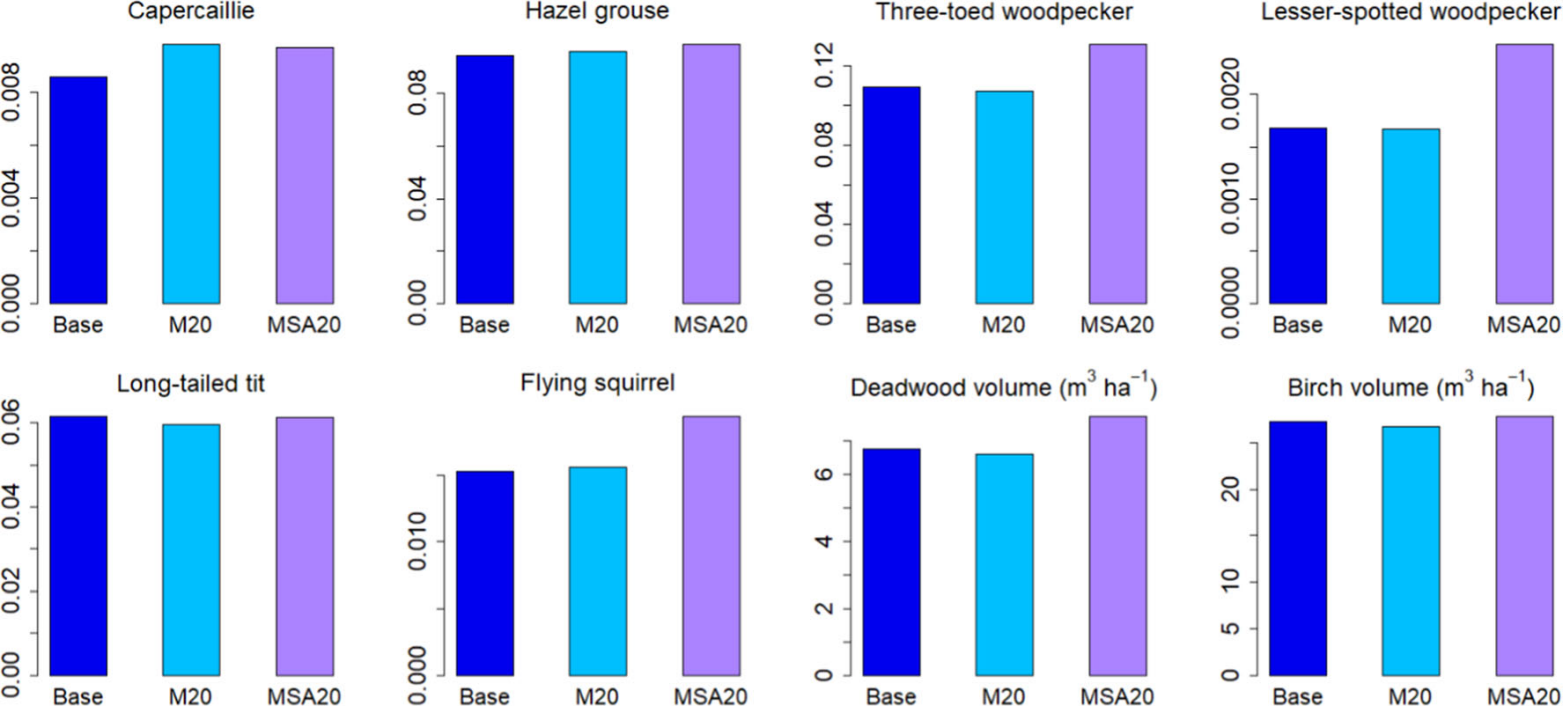
Alternative managements according to forest owners’ preferences at end of simulation period (2050). Means are taken over current managed area. M20 = 80% base + 20% mitigation scenario, MSA20 = 80% base + 14.8% mitigation scenario + 5.2% set-aside

In a comparison of the two mixed scenarios devised on the basis of forest owners’ preferences, we again found no differences in the C balance variables but some impacts were detectable on the biodiversity indicators. Increasing indicator values could be detected in four cases for the combined 20% mitigation and set-aside scenario (capercaillie, lesser-spotted woodpecker, flying squirrel, deadwood volume) and in one case for the 20% mitigation scenario (capercaillie) (Fig. 8).

**Fig. 9:**
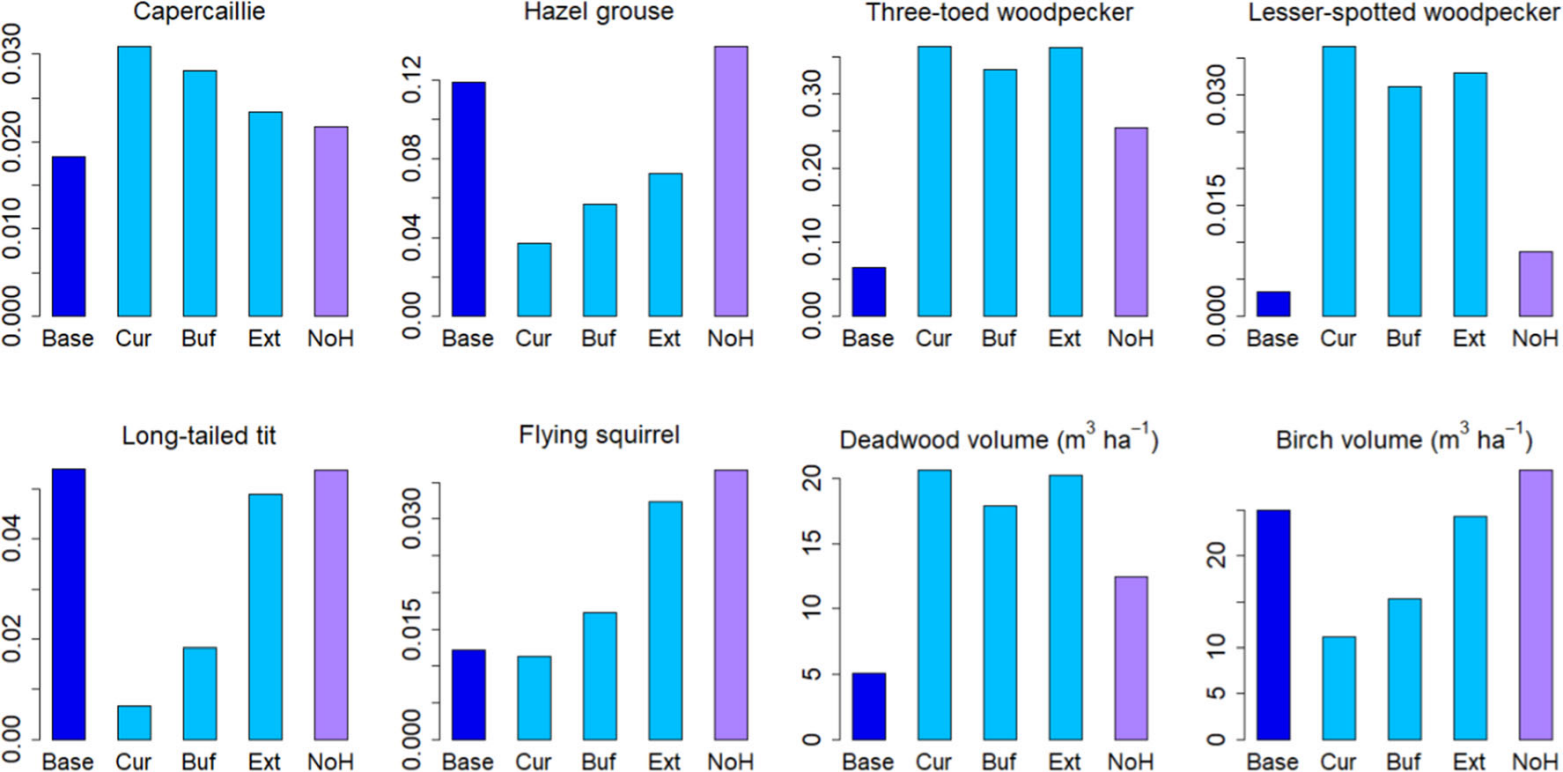
Biodiversity indicators in managed and unmanaged stands—period mean per hectare. Light blue bars show the mean suitability in protected stands under each protection strategy. Dark blue bar gives the same mean for unprotected stands in Base management strategy and under current harvest level (BAU). The purple bar gives the mean suitability across currently managed areas set aside in 2021 following the NoHarv scenario. *Cur* current protection areas, *Buf* current areas and their buffer zones added in protection scenario, *Ext* extended protection areas, at least 10% in each region, *NoH* NoHarv scenario for currently managed areas

Since increasing the protected area led to increased values of biodiversity indicators in both management-driven and demand-driven scenarios, it is interesting to take a closer look at the indicators in protected areas under the different protection scenarios simulated (Fig. 9). For half of the indicators (capercaillie, three-toed woodpecker, lesser-spotted woodpecker and deadwood), current protected areas provided a higher quality habitat than managed forest. For the other half, habitat suitability was on average the same or higher outside protected areas. However, in all such cases additional set-aside areas (buffers and extension to at least 10% regionally) increased the average suitability, indicating that the poor performance of current protected areas might reflect unfavourable placement of current protection or alternatively, limited amount of optimal habitat (e.g., large deciduous forests; see Supplementary Information 3) to these species in current protected areas.

Furthermore, differences were found between the different protected area strategies, including (1) current protected areas (8.5%), (2) current protected areas + buffer zones (11.5%), (3) extension to at least 10% regionally, and (4) the set-aside achieved using the NoHarv scenario in the forest owners’ preference scenario (additional 5.2%) (Fig. 9). These differences were not always in the same direction, but notably, the set-aside (NoHarv) areas reached the highest values of all conservation strategies in four cases out of eight (hazel grouse, long-tailed tit, flying squirrel, birch volume). In the rest of the cases, all protected areas (current, current + buffer, extended) outperformed the managed forests and the set-aside (NoHarv).

## Summary

**Fig. 10:**
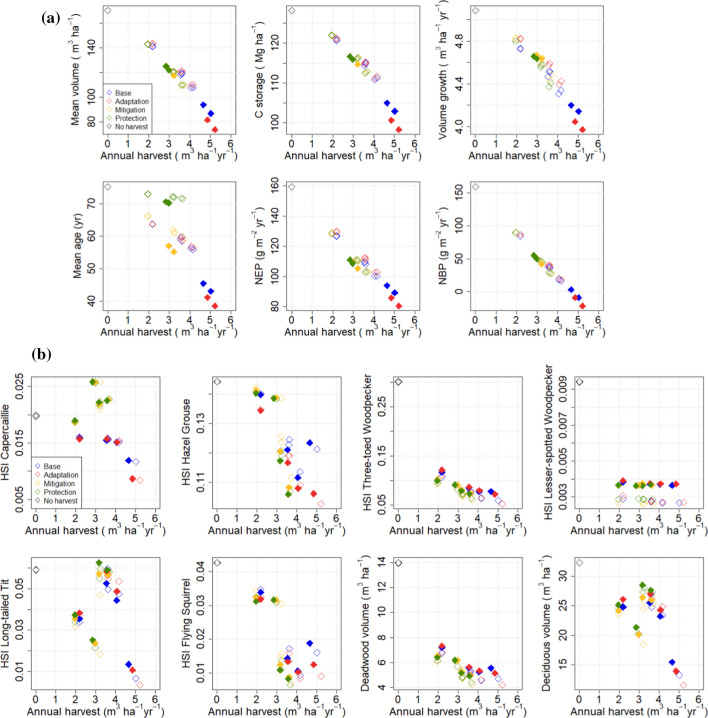
**a** Temporal mean values (2021–2050) of national mean carbon balance indicators in all management-driven (filled symbols) and demand-driven (open symbols) scenarios. **b** Temporal mean values (2021–2050) of national mean biodiversity indicators in all management-driven and demand-driven scenarios. Filled symbols are with extended protected area (at least 10% in all regions, 13.7% nationally), open symbols are with current protected area (8.5% nationally). In the symbols in **a **and **b**, colour indicates management strategy (legend in figure) and each have one free and three constrained harvest levels

The above analyses can be summarized by plotting the temporal average C balance and biodiversity indicators against the temporal average harvest level for the period 2021–2050 (Fig. 10a, b). There is a strong correlation between the mean harvest level and the indicator values for the C balance indicators. The management-driven scenarios follow the same pattern, although the harvest level was a consequence, not a constraint, for cuttings. The same pattern exists although less clearly for the biodiversity indicators, with the most evident trends being manifested with those indicators that attain their highest values under the NoHarv scenario.

## Discussion

### The role of harvest level and management strategy for C balance and biodiversity in managed forest (RQ1, RQ2)

The central finding of this study was that harvest level was key to carbon stocks and fluxes, regardless of any other management actions taken, whereas the biodiversity indicators considered were also sensitive to the forest structural attributes related to the management actions (Fig. 10). This result was independent of whether we applied the management-driven or demand-driven scenarios.

The result that harvest level is an essential determinant of the national forest C balance has been obtained in previous studies that did not consider other methods of managing stand structure (e.g., species, retention trees, deadwood) (Härkönen et al. 2019; Kalliokoski et al. 2020). Studies at management unit level have found that management options are significant for the wood production (Díaz-Yáñez et al. 2019; Duflot et al. 2022) or C balance of the management unit considered (Eyvindson et al. 2018; Blattert et al. 2022). This is similar to our results for the management-driven scenarios which produce very different C balance indicators for the different strategies (Fig. 2). However, they also generate very different cutting levels (Fig. 4) and thus follow the same harvest-dependent pattern as the demand-driven scenarios (Fig. 10a). This follows from the fact that in this study, like in most management impact studies (Eyvindson et al. 2018; Díaz-Yáñez et al. 2019; Blattert et al. 2022; Duflot et al. 2022), rotation length and thinning interval were key components of the management strategies, and they are crucial in determining the steady-state harvest level (Heaps 2015).

The additional determinants of management strategies included fertilization (for the adaptation strategy), not collecting harvest residues (mitigation and protection), choice of species at regeneration and thinning (adaptation and protection), choice of harvested stands based on their age (mitigation and protection), leaving retention trees (protection), and leaving protected buffer zones (protection). Two of these, fertilization and harvest residue collection, also operated through harvest intensity: fertilization accelerated growth, bringing the stands faster to harvest maturity, and leaving the harvest residues at sites reduced the total harvest, although the round wood harvests remained the same (Fig. 5). The rest of the measures had implications on forest structure (mean age, species composition, dead wood), which did develop differently under the different management strategies but did not reflect on the national C balance at least over the time period considered.

In contrast, the structure variables were important for the biodiversity indicators calculated here, rendering them less dependent on the harvest level than the C balance indicators (Fig. 10). As a result, some of the indicators were clearly management dependent and others depended on the extent of protected area (Fig. 10b). The most clearly management dependent indicator, capercaillie, described the suitability of a stand as capercaillie lekking site and was assigned high values for stands with pine and spruce mixture and relatively low overall density (Mönkkönen et al. 2014). The indicator for lesser-spotted woodpecker, on the other hand, was defined high in old stands with a large amount of deciduous dead wood (Mönkkönen et al. 2014), and therefore reached its maximum values in protected forests, independently of harvest level or management strategy (Figs. 9 and 10b). The indicator for Siberian flying squirrel which required a spruce-dominated forest with deciduous mixture (Mönkkönen et al. 2014) showed its highest values under the low-harvest scenario and in the management-driven mitigation and protection strategies that implied reduced harvests (Figs. 3 and 7).

The direct mass-based variables used here as biodiversity indicators are also reflected in the indicators that depend on them, i.e., dead wood volume (three-toed woodpecker, lesser-spotted woodpecker) or deciduous volume (hazel grouse, long-tailed tit, flying squirrel). We found a strong dependence of dead wood volume on the harvest level, whereas deciduous volume depended somewhat on the management strategy. These are factors that could be more strongly affected through management recommendations, e.g., through a requirement to leave more deadwood and live deciduous trees, particularly large or old individuals, at harvests, and to favour deciduous trees at regeneration more strongly than was done here (Oettel and Lapin 2021). According to the present results and also supported by empirical analysis (Asbeck et al. 2021), this could increase the value of biodiversity indicators without affecting the C balance.

### Implications of extended protected area (RQ3)

The present analysis of extended set-aside under the original harvest levels suggests that the current state of the Finnish forests would allow for a moderate extension of the strictly protected areas without affecting the round wood supply at the national level, although some re-allocation of the relative regional harvest levels would help maintain the long-term harvest potential. If moderate climate change increased forest productivity, as expected  (e.g. Junttila et al. 2023), and if allocation of harvests was optimized (Blattert et al. 2022), the potential for set-aside could be considerably larger.

In contrast to the unaffected C balance, mean values of most of the biodiversity indicators were raised by the extension of the protected forest area (Fig. 7). Part of this was likely due to the fact that the requirement of at least 10% of protected area per region meant a large increase in unmanaged forest in the south of the country where some of the indicators reached high values. Particularly, these included long-tailed tit, Siberian flying squirrel and birch volume, indicators that reached their highest values in the extended protected areas. Deadwood volume and the two woodpecker species showed high values in all protected areas, while indicators of capercaillie and hazel grouse were less clearly affected by either no-harvest or protection areas. These results were further demonstrated in our example where we employed either alternative management or a mixture of management and set aside in 20% of the current managed forest, the latter yielding a small but detectable increase in the national mean biodiversity indicators (Fig. 8).

### Comparison of management-driven and demand-driven approach (RQ1, RQ2)

Our “management-driven” simulations investigated the hypothetical situation where all forest owners strictly follow assumed management strategies. This approach is commonly taken in studies analysing an individual forest owner’s options from the point of view of optimizing their objectives, whether economic or value-based (Eyvindson et al. 2018; Díaz-Yáñez et al. 2019; Blattert et al. 2022; Duflot et al. 2022). In the longer term, each strategy would determine their own average harvest level, including thinnings and clear cuts (e.g., Blattert et al. 2022), and the system would approach a steady age-distribution generated by this (Heaps 2015). However, if the initial age distribution is far from this steady state, there will be overshoots, such as the large cuttings observed in the first year of our simulation (Fig. 4a), caused by the high proportion of stands older than the recommended rotation length (Fig. 2). After the initial harvest shock the cutting levels will gradually approach the mean annual increment as the age distribution approaches its steady state (Fig. 4a). Before that, the harvest levels will either increase or decrease, depending on the average rotation length relative to its current value (Fig. 10a, b). This suggests that the frequent advice from forest professionals to forest owners, to carry out all management actions on time, may not be strictly applicable. In practice, many other drivers, including wood demand and forest policies, are behind the management decisions (Eyvindson et al. 2018; Blattert et al. 2022).

The “demand-driven” simulations, assuming an externally given “demand” or harvest level, perhaps more realistically describe the development of the national growing stock under harvests, and therefore that of the related carbon and biodiversity indicators. We emphasize, however, that no optimization was applied in allocating the harvests to different stands when the supply of stands mature for harvest or thinning was greater than the harvest level, but a random selection of stands was applied. For comparison, the Finnish national-scale forest simulation system MELA (Lappi 1992; Hynynen et al. 2002), allocates cuttings based on a routine optimizing the economic returns for clusters of inventory plots (termed “management unit”). The total annual cut is determined from this optimization but not without further constraints, such as percentage of area undergoing thinnings or clear cuts (Ministry of Agriculture and Forestry 2019) or maximum annual change in total harvest (Hirvelä et al. 2017). On the other hand, general partial equilibrium models (Northway et al. 2013) determine the harvest level as a balance between supply and demand, where the supply can be constrained by the growing stock and management rules, and the demand is either global, continental or national, with constraints for the external part of the demand (Solberg et al. 2003; Kallio et al. 2013, 2016). From the point of view of an individual country the results of this can appear very similar to the externally determined harvest level of this study (Härkönen et al. 2019).

From the perspective of these results, the alternative management strategies devised by the stakeholder meeting seem relatively less important than the total harvest level for securing the goals expressed for the strategies. Nevetheless, the indicators of C balance and biodiversity appear to develop more favourably with the mitigation and protection strategy than with the other two strategies (Figs. 2, 3, 6 and 7). We note that the mitigation strategy was here defined as “mitigation through forest C sequestration” (Sect. "Management strategies and cutting levels".) which obviously only covers part of all climate change mitigation through forest management. A more complete picture including industrial carbon fluxes combined with the present analysis is provided by Forsius et al. (2023). Other parallel studies in Finland have considered the potential role and economy of continuous-cover forest management on reducing peatland emissions at site level (Nieminen et al. 2018; Shanin et al. 2021), and studies are ongoing to evaluate its impacts at country scale. Planned future work with our approach also includes incorporating the effects of aerosols and albedo in the analysis.

A strong rationale for the adaptation strategy expressed by the stakeholder meeting was to reduce the potential damage caused by the increasing occurrence of disturbances expected under climate change, using shorter rotations and more intensive thinnings as a means, as also recommended nationally (Äijälä et al. 2019). Although this clearly implied less carbon sequestration and lower biodiversity values than the other strategies in this study, the result could have been different had we been able to simulate the occurrence of forest disturbance. Although disturbance clearly threatens the growing stock and thus the forest owners’ potential income (Valsta 1992; Xu et al. 2016), literature on the effects of natural disturbance on forest carbon budgets and biodiversity is not conclusive. Thom and Seidl (2016) found in a thorough literature review that while disturbances generally reduced ecosystem services, they simultaneously enhanced biodiversity, whereas the review by Mikoláš et al. (2021) presented evidence that disturbance had positive effects on both biodiversity and carbon sequestration.

### Study limitations and future directions

Like in all modelling studies, our results are conditional on the model and assumptions used. The PREBAS model was developed for analysing forest growth and C balance, and that is what previous model calibration and testing has focused on (Minunno et al. 2019; Mäkelä et al. 2020). Here, we introduced modified management actions and previously published biodiversity indicators to allow for a more extensive analysis of management impacts. Because PREBAS describes stands based on species mean trees, it is presently not able to account for more complex management-dependent structures that could be significant especially for biodiversity. We therefore expect that the simulations underestimated the management sensitivity of biodiversity indicators. The results could also have been more distinct if a higher threshold value had been chosen for the indicators (e.g., Duflot et al. 2022 used 0.7 instead of the 0.5 used here). Kujala et al. (2023) demonstrated that the most suitable habitats were the least sensitive to input and parameter uncertainties and would therefore minimize the impact of any random factors on the result. That we nevertheless were able to capture such sensitivity to management with our simplified model suggests that structural attributes indeed play an important role in safeguarding biodiversity.

Model structure also confined our description of the alternative management actions. For example, we were not able to manage stands to increase the natural size variability of trees in stands, or by introducing new, non-native species or genetically improved forms that would be better suited for future conditions (Äijälä et al. 2019). Neither our model nor the input data were able to incorporate information about the occurrence of individual trees regarded crucial for biodiversity, such as old, large aspen (Kivinen et al. 2020). More importantly, our model could so far not be used for simulating continuous cover as a management option, which in some studies has been found to be the most sustainable forest management method (Eyvindson et al. 2021; Blattert et al. 2022). Although continuous cover harvests are reported to be only ca. 3% of all harvests (Finnish Forest Centre 2020), they are likely increasing in the future. We can conclude from the present results that for the C balance, the harvest level should be the decisive factor for continuous cover as well. Other studies have indicated that continuous cover forest structure would be desirable for some indicator species, while others would benefit more from even-aged stands (Duflot et al. 2022).

One potential problem with the type of biodiversity indicators used here is that they were treated at the stand level only, dependent on the respective stand structure variables. This ignores any impacts of landscape and larger level factors on biodiversity, such as connectivity of habitats and other interactions between neighbouring stands (Virkkala et al. 2022; Kujala et al. 2023). In addition, the biodiversity indicators do not account for climatic or other environmental factors, such as presence of other land use activities, shown to be relevant in determining habitat suitability for some of these species (Virkkala et al. 2022). We nevertheless note that the relationships between the species and stand structural variables are well studied and stand variables have shown higher importance for biodiversity indicator forest bird species than landscape factors (Virkkala et al. 2022). Moreover, all of our indicator species are ecologically well-known and most of them have also been validated with field observations in different studies (Jansson and Angelstam 1999; Reunanen et al. 2004; Hurme et al. 2007; Roberge et al. 2008). All of the HSI index models are also nonlinear, indicating that the structural variable values themselves do not directly translate into habitat suitability but the HSI models provide additional information on the goodness of the forest stand for these biodiversity indicators.

Due to the between-species ecological differences there is no general habitat suitability metric that could be used to provide optimal conditions for all indicator species. Instead, a combination of different habitat types may provide a more sustainable solution (Duflot et al. 2022), although some indicator species have a higher capacity to reflect the overall spatial variation of threatened forest species than others. In our case, three-toed woodpecker can be considered as a useful indicator for many threatened old-growth forest species while capercaillie and hazel grouse, due to their preference towards younger mixed forests, are linked to fewer threatened species. Nevertheless, the current analysis has provided corroborating evidence to the hypothesis that forest structure variables important for biodiversity can be controlled by forest management and are relatively independent of carbon stocks and fluxes in the forests. A more in-depth analysis including the spatial co-distribution of biodiversity and carbon-based indicators is given by Kujala et al. (2023).

Regarding the carbon balance analysis, the present study does not consider the dependence of ground water level on harvests in drained peatlands, which may become important especially if the harvests differ a lot from the current normal practice. Secondly, in relation to the modified management actions in upland forests, we did not consider the possibility that fertilization increases the potential to sequester carbon in soils, found in some empirical studies (Zhao et al. 2022), nor the possible interactions of fertilisation and biodiversity especially in the poorest forest sites that contain some of the most threatened forest habitat in Finland (Strengbom et al. 2011). As a whole, a proper process-based description of soil carbon and nitrogen interactions is still under development for the PREBAS model. On the other hand, our model included ground vegetation as a carbon pool, which has not been the case in many other analyses, although ground vegetation components have been included as non-wood forest products (e.g. Blattert et al. 2022).

## Conclusion

In this study we analysed the combined impact of prescribed forest management rules and country-wide harvest levels on carbon balance and biodiversity related indicators in Finland. In our results, harvest level was key to carbon stocks and fluxes, regardless of any additional management actions taken and allowing for a moderate extension of strictly protected forest. The same trend with harvest levels was detectable to some extent in the biodiversity indicators, but these were also sensitive to management strategies and showed overall higher national average values under increased set aside even at unchanged harvest levels. This suggests that although the current and even slightly higher cutting levels could be sustainable from the perspective of wood production, higher yields also mean more adverse effects on carbon sequestration and biodiversity. According to our results, there is some potential to increase biodiversity even under the current harvest intensity, both through directed management actions and through extension and proper allocation of protection areas. However, reduced harvests are needed to guarantee that the forests remain carbon sinks in the next few decades.

### Supplementary Information

Below is the link to the electronic supplementary material.

Supplementary file1 (PDF 2175 kb)

## Footnotes

1 http://www.ibccarbon.fi/en-US.
