# Supplementary material for: Effect of forest management choices on carbon sequestration and biodiversity at national scale
Source: Ambio. 2023 Aug 3;52(11):1737–56. doi: 10.1007/s13280-023-01899-0 (PMC10562327; doi:10.1007/s13280-023-01899-0)
Supplement: Supplementary file 1 — Supplementary file1 (PDF 2175 kb) [file 13280_2023_1899_MOESM1_ESM.pdf]

Ambio
Electronic Supplementary Material
This supplementary material has not been peer reviewed.

Title: **Effect of forest management choices on carbon sequestration and biodiversity at national scale**

Authors: Annikki Mäkelä, Francesco Minunno, Heini Kujala, Anna-Kaisa Kosenius, Risto K. Heikkinen, Virpi Junttila, Mikko Peltoniemi, Martin Forsius

Contents

S1. Mortality model
S2. Ground vegetation model
S3. Habitat suitability index
S4. Age class distribution
S5. Stakeholder meeting
S6. Results

S1. Mortality model

The stand-level mortality models developed by Siilipehto et al. (2020) where implemented in PREBAS. The models were developed using permanent national forest inventory data from Norway, Sweden and Finland. The models are logistic functions that predict mortality in two steps: the first model predicts the probability of survival (M_1), the second model predicts the proportion of basal area that remains after a mortality event (M_2).

The structure of the model is the following:

$$P = (1 + \exp(-X'b)) \quad \text{(S1.1)}$$

where P is the probability of no mortality (in M_1) or the proportion of survived trees (in M_2); $X'b$ is the linear combination of predictor variables times the parameters of estimated in Siipilehto et al. (2020).

The parameters and predictor variables of M_1 are reported in Table 5 of Siipilehto et al. (2020). Similarly, the details of the linear part $X'b$ for the proportion of basal area in surviving trees is reported in Table 6 of Siipilehto et al. (2020).

Mortality is then estimated using in combination the two models:

$$M_G = \left(1 - P(M_1)\right)\left(1 - P(M_2)\right)G \quad \text{(S1.2)}$$

where M_G is the basal area of the dead trees and G is the stand basal area of the growing stock.

The mortality model described above was applied only to the protected areas, jointly with the Reineke mortality model (Reineke, 1933), at annual time step. In the managed forests, only the Reineke function was used (Minunno et al., 2019).

References

Minunno F., Peltoniemi M., Härkönen S., Kalliokoski T., Mäkinen H., Mäkelä A. 2019. Bayesian calibration of a carbon balance model PREBAS using data from permanent growth experiments and national forest inventory. *Forest Ecology and Management* 440: 208-257. https://doi.org/10.1016/j.foreco.2019.02.041

Reineke, L. 1933. Perfecting a stand-density index for even-aged forests. *Journal of Agricultural Research, 46*, 627-638

Siipilehto J., Allen M., Nilsson U., Brunner A., Huuskonen S., Haikarainen S., Subramanian N., Antón-Fernández C., Holmström E., Andreassen K., Hynynen J. (2020). Stand-level mortality models for Nordic boreal forests. Silva Fennica 54 no. 5 article id 10414. https://doi.org/10.14214/sf.10414

S2. Ground vegetation model

Overview

The purpose of the ground vegetation (GV) module is to estimate the contribution of ground vegetation to the modelled ecosystem carbon fluxes, including (1) photosynthesis, (2) respiration, and (3) litter fall (Figure S1). We first estimate the percentage cover of different functional groups of ground vegetation, then convert this to above-ground and below-ground biomass using conversion equations. Turnover can be related to biomass, and further, with the simplifying assumption that ground vegetation is approximately at steady state, growth and respiration can also be approximated.

There is established empirical evidence that the percentage covers of different GV types depend on site fertility (Tonteri et al. 2005) and light reaching the bottom layer (Strengbom et al. 2004), which vary with the development stage of the stand (Tonteri et al. 2005). The PREBAS GV module therefore uses site fertility class and the proportion of light absorbed by the tree canopy as explanatory variables of the percentage cover of ground vegetation types.

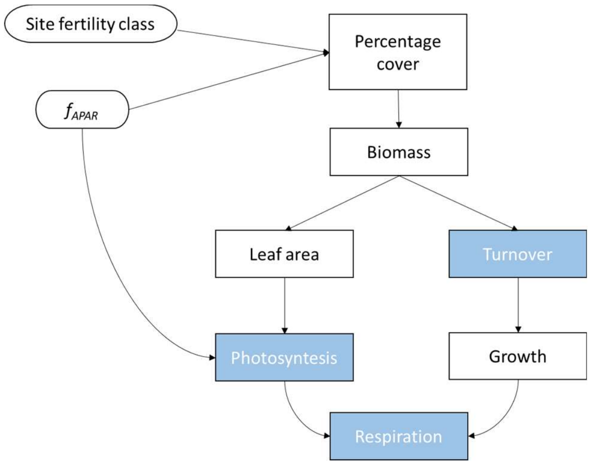

Figure S2.1. Flowchart of the calculation model. Inputs are site fertility class and proportion of par radiation not absorbed by the tree canopy f_{APAR}, and outputs are ground vegetation photosynthesis, turnover and respiration.

Model formulation

Percentage coverage

The core of the GV module is the model of percentage covers of different functional groups of GV. In order to keep the model simple, we only use three groups, some of which are combinations of groups in a more detailed classification based on the light and nutrient demand of different plant types: (1) grasses and herbs (x_g), (2) shrubs (x_s), and (3) mosses and lichens (x_m). The models are based on a qualitative understanding of the light dependence of the groups, with level parameters determined separately for different site fertility classes (Tonteri et al. 2005). Thus, we assume that grass and herb coverage increases linearly with the light

entering the understorey, whereas the percentage coverage of mosses and lichens decreases with increasing light. Shrubs are assumed to reach a maximum percentage coverage at intermediate light levels and minima at full light and no light. These dependencies are described by the following equations:

$$x_g = a_g(1 - f_{APAR}) + b_g$$
$$x_s = a_s(f_{APAR} + 0.2)\left[\ln\left(\frac{1}{f_{APAR}}\right)\right]^{0.5} \qquad \text{(S2.1)}$$
$$x_m = a_m(1 - f_{APAR}) + b_m f_{APAR}$$

where the dependence on site fertility is thought to be embedded in the parameters (a_g, b_g, a_s, a_m, b_m).

The parameters were evaluated through a heuristic procedure where empirically estimated country-level mean values per site class and age class were predicted with Eqns (S2.1) using a PREBAS-model-based average age dependence of f_{APAR} to connect the age classes and model outputs (Table S1, Figure S2.2, S2.3).

Table S2.1. Parameters for model (S2.1).

Parameter	Grove	Grove-like	Herb-rich	Sub-xeric	Xeric
a_g	0.4	0.3	0.3	0.1	0.05
b_g	0.4	0.3	0.1	0.05	0
a_s	0.1	0.3	0.55	0.65	0.7
a_m	0.1	0.1	0.3	0.6	0.6
b_m	0.3	0.4	0.6	0.8	0.8

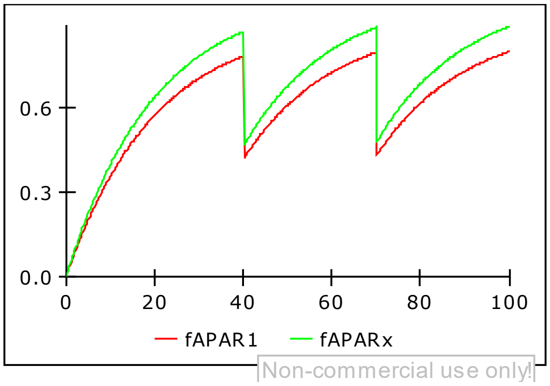

Non-commercial use only!

Fertility class	$f_{\max}$	Typical dominant species
grove	0.95	deciduous, spruce
grove-like	0.9	spruce
herb-rich	0.9	spruce, pine
sub-xeric	0.8	pine
xeric	0.7	pine

Figure S2.2. Assumptions about the mean relationship between stand age and f_{APAR} used in heuristic parameter estimation for percentage ground cover (Eqn S1). The left-hand side shows the mean temporal development of f_{APAR}, following the basic equation $f_{APAR} = f_{\max}(1 - e^{-0.05t})$ interrupted by thinnings at times 40 and 70 yrs. The table on the right gives the maximum f_{APAR}, $f_{\max}$, for the different site fertility classes used.

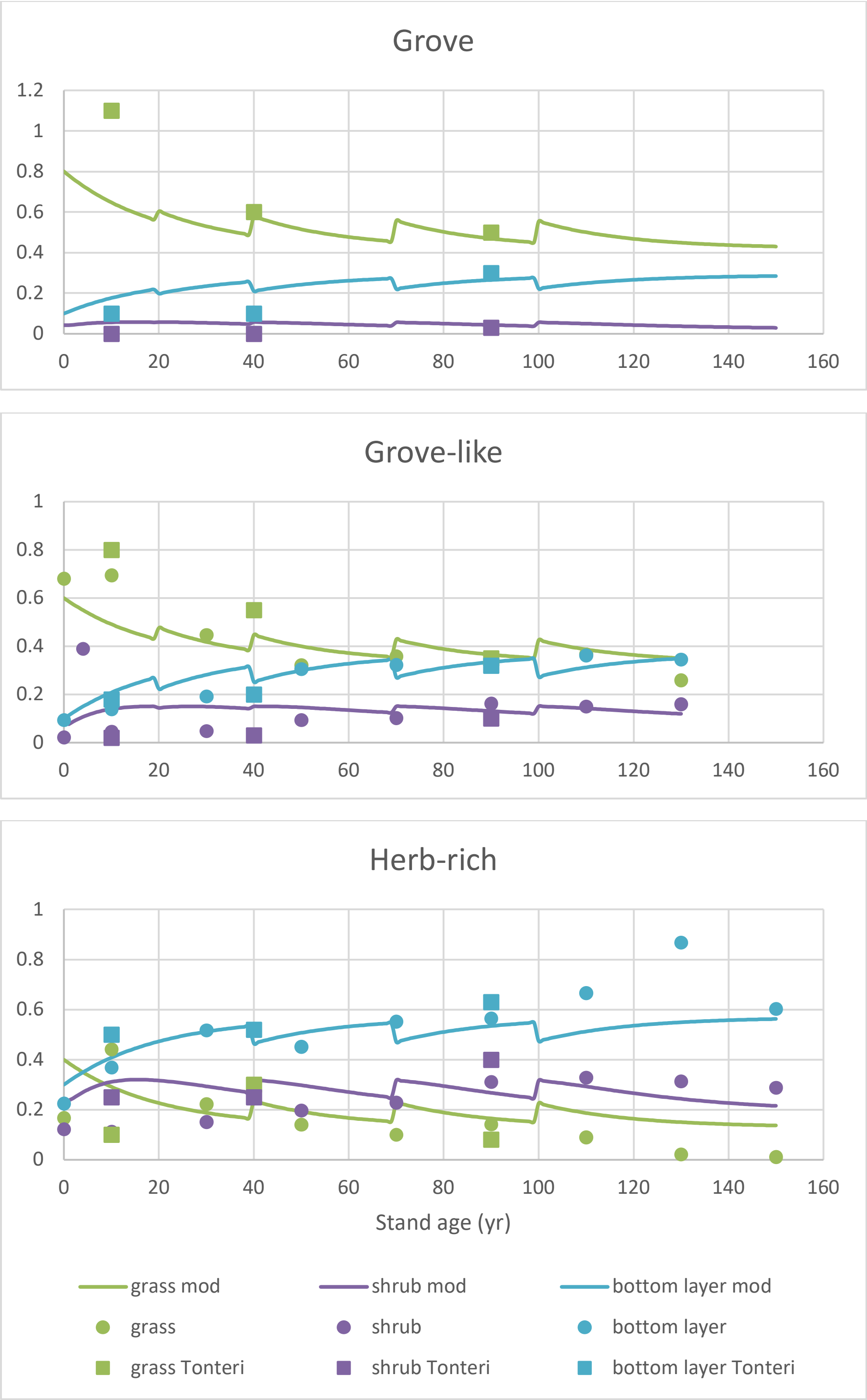
Grove
1.2
1
0.8
0.6
0.4
0.2
0
0
20
40
60
80
100
120
140
160
Grove-like
1
0.8
0.6
0.4
0.2
0
0
20
40
60
80
100
120
140
160
Herb-rich
1
0.8
0.6
0.4
0.2
0
0
20
40
60
80
100
120
140
160
Stand age (yr)
grass mod
shrub mod
bottom layer mod
grass
shrub
bottom layer
grass Tonteri
shrub Tonteri
bottom layer Tonteri

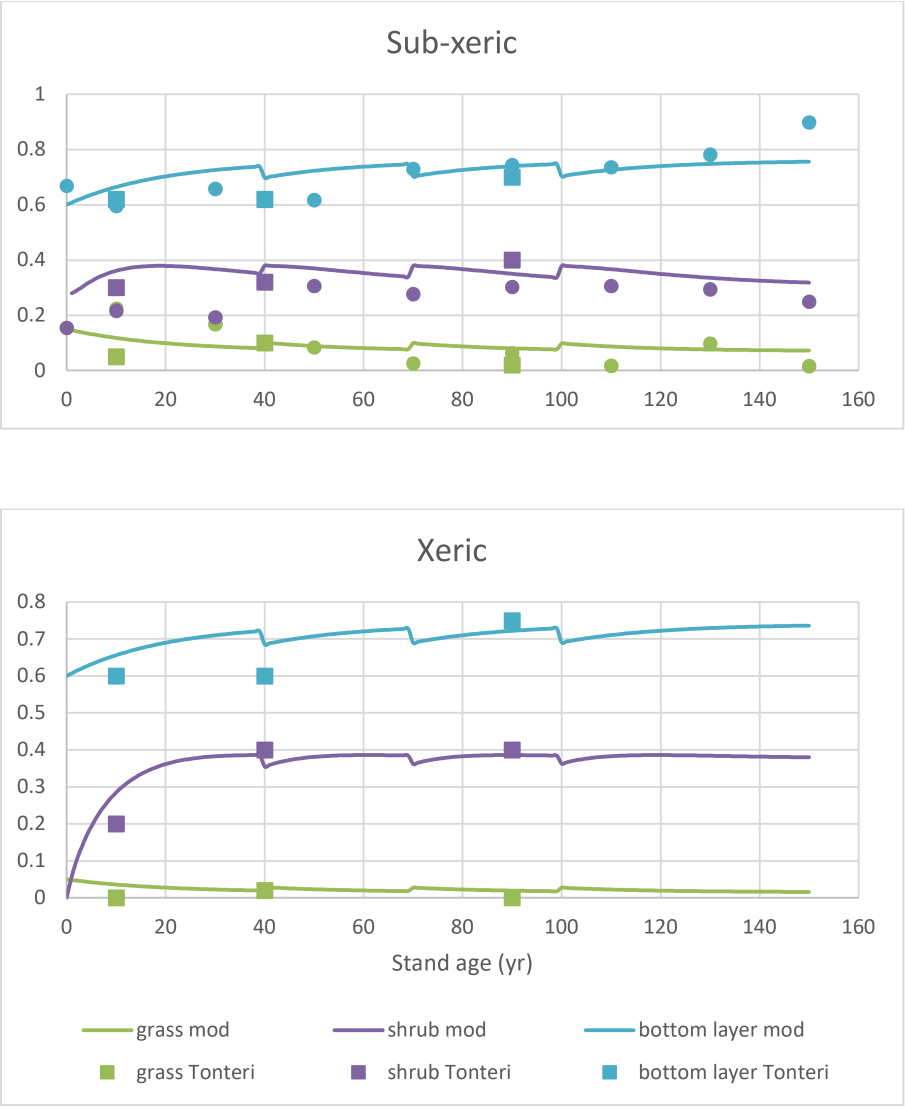

Figure S2.3. Measured and modelled percentage coverages as a function of stand age. The observations come from Tonteri et al. (2005) (squares) and Tähkälä (2011) (circles). Tonteri et al. (2005) presented a summary of ground vegetation percentage cover in Finland based on 3000 measurement points of the 8th Finnish National Forest Inventory (NFI) 1985-1986. The data were described by classes of 5 vegetation zones (hemiboreal, south, central and north boreal, northern Lapland), five site fertilities, three dominant species, and three forest development stages (regeneration, young stand, mature stand). Tonteri (2011) used a subsample of data in Tonteri et al. (2005) for a more detailed analysis. Her data set covers three fertility classes, including 180 sub-xeric, 423 herb-rich and 251 grove-like sites on upland soils in southern Finland. Tähkälä (2011) analysed the data with respect to stand age, stand basal area and time from the latest harvest (either clear cut or thinning). A general feature of the data was that the variability was very high, the standard deviations being approximately equal to the means in all analyses. We used the results from Tähkälä (2011) as our primary source but extended the results from three to five fertility classes using Tonteri et al. (2005).

Biomass, turnover and leaf area

Based on empirical findings (Muukkonen et al. 2006, Lehtonen et al. 2016), we assumed an exponential relationship between percentage cover of species group i and mass per unit area. In order to determine leaf area and turnover rates, the mass was divided into above-ground (m_i^a) and below-ground (m_i^b) components:

$$m_i^a = \alpha_i^a x_i^{\beta_i^a}$$
$$m_i^b = \alpha_i^b x_i^{\beta_i^b} \qquad \text{(S2.2)}$$

where α_i^a, β_i^a, α_i^b and β_i^b are parameters for the above-ground and below-ground components, respectively (Table S2.2). The parameter values were obtained by simplifying the mixed model equations estimated by Lehtonen et al. (2016) separately for grasses, herbs, shrubs, mosses and lichens. Here we combined the original functional groups of grasses + herbs and mosses + lichens. This was done by first calculating the mass estimates for the original classes, then fitting the above simplified equations to the combined classes.

Compartment specific turnover rates were obtained from Lehtonen et al. (2016), again by combining original classes when needed. This assumed constant annual turnover rates for all above-ground and below-ground biomass components, such that turnover T_i^j ($i \in (g, s, m); j \in (a, b)$) is

$$T_i^j = s_i^j m_i^j \qquad \text{(S2.3)}$$

with s_i^j denoting the component-specific turnover rate (yr^{-1}).

Table S2.2. Parameters for different plant types. α_i is expressed in kg DW ha^{-1} and turnover rate is yr^{-1}.

**Plant type**	**a / b**	$\boldsymbol{\alpha_i}$	$\boldsymbol{\beta_i}$	$\boldsymbol{\lambda_i}$	$\boldsymbol{s_i}$
Shrubs South Finland	a	3152.4	1.107	6 m^2 / kg	0.37
Shrubs South Finland	b	5016.0	0.831		0.08
Shrubs North Finland	a	3627.1	0.948	6 m^2 / kg	0.37
Shrubs North Finland	b	5587.7	0.45		0.08
Grasses and herbs	a	1390.1	0.9496	7 m^2 / kg	0.54
Grasses and herbs	b	1278.3	0.6628		0.59
Mosses and lichens	a	1637.1	0.8289	1 m^2 / kg	0.20

Leaf area L_i, was assumed to be obtained from the above-ground biomass through multiplication with specific lear area, λ_i (m^2 kg^{-1}):

$$L_i = \lambda_i m_i^a \qquad \text{(S2.4)}$$

The specific leaf area of shrubs and grasses + herbs was based on Palmroth et al. 2014. They estimated total biomass, root to shoot ratio and foliage to biomass ratio of bilberry and cowberry in Rosinedal pine forest, northern Sweden. We used this information to estimate foliage area per above-ground biomass, which is shown in Table S2.2 as $\boldsymbol{\lambda_i}$. We estimated the value for grasses and herbs as somewhat higher than that for shrubs. The leaf area for mosses and lichens is a guess that produces approximately the share of photosynthesis of mosses and lichens given by Laine et al. (2016).

Contribution to ecosystem GPP and respiration

For PREBAS, we add the LAI of GV to forest canopy LAI and use this as forest ecosystem LAI. This is used in estimating ecosystem and canopy GPP in PREBAS (Peltoniemi et al. 2015). With the above parameter values, the model gives an almost linear relationship with tree canopy f_{APAR} and the combined proportion of absorbed radiation, $f_{APAR,tot}$ (Figure S2.4).

The contribution of GV to canopy respiration is estimated using the assumption that GV is approximately at steady state, such that annual growth equals annual litter fall. This allows us to estimate GV respiration as

Respiration(GV) = GPP(GV) - Litter fall(GV)

where GPP and litter fall are evaluated as explained above.

Because the GV model is not dynamic, but GV biomass is determined by the current value of f_{APAR}, the above equation may lead to negative respiration at times of thinning or clear cuts. This is avoided by restricting respiration to non-negative values.

Model evaluation

Given that the core of the model is based on phenomenologically justified equations (Eqn S2.1) with non-systematic parameterization, we wanted to evaluate its performance against independent studies. Two suitable studies were found: Kulmala et al. (2011), Tupek et al. (2016).

Kulmala et al. (2011) presented data from an age series of five pine stands representing herb-rich or sub-xeric sites in southern Finland. They also reported the canopy all-sided leaf area, which can be used here to test our results. We use $k = 0.18$ for the canopy (Duursma and Mäkelä 2007) to obtain the respective f_{APAR} values. We calculate the contribution to total f_{APAR} of the ground vegetation as follows:

$$f_{APAR,tot} = f_{APAR} + \frac{P_{GV}}{P_0} \qquad \text{(S2.5)}$$

where f_{APAR} is the proportion of light absorbed by the tree canopy and P_{GV} is total photosynthesis by ground vegetation, both as estimated by Kulmala et al. (2011), and we use $P_0 = 1200$ g m^{-2} yr^{-1} (Minunno et al. 2016).

A comparison of the above-ground biomass of ground vegetation reported by Kulmala et al. (2011) and the proposed model with sub-xeric sites shows that in this case, the model underestimates shrub biomass but slightly overestimates the other biomasses (Figure S2.4a). This could be indicative of the wide variability of ground vegetation between individual stands. Nevertheless, the predicted contribution of GV to $f_{APAR,tot}$ was very much in line with that calculated from Kulmala's observations (Eqn S2.5) (Figure S2.4b). This relationship appears independent of site fertility class in the model.

a)

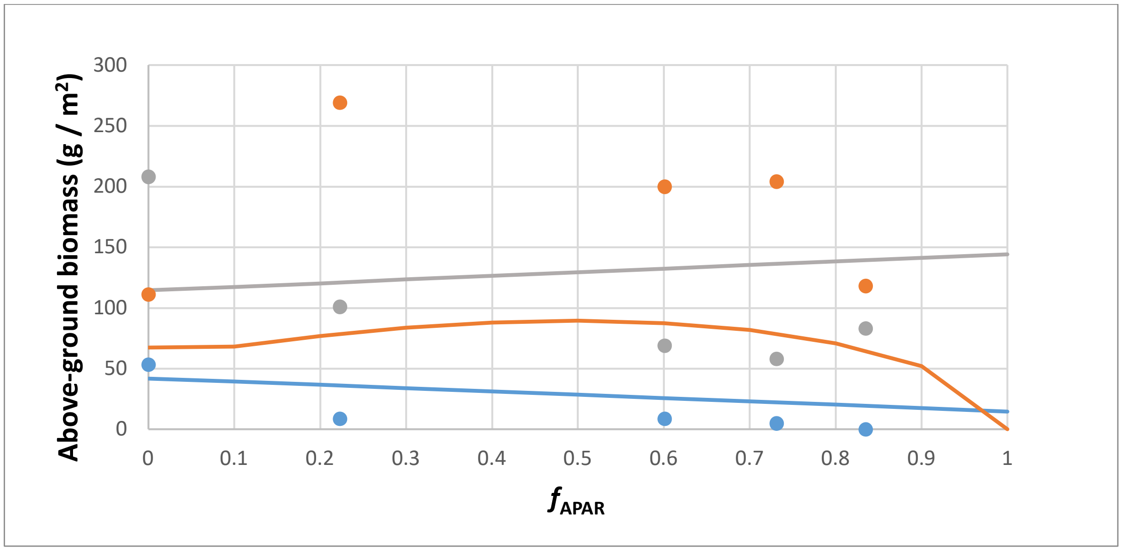

b)

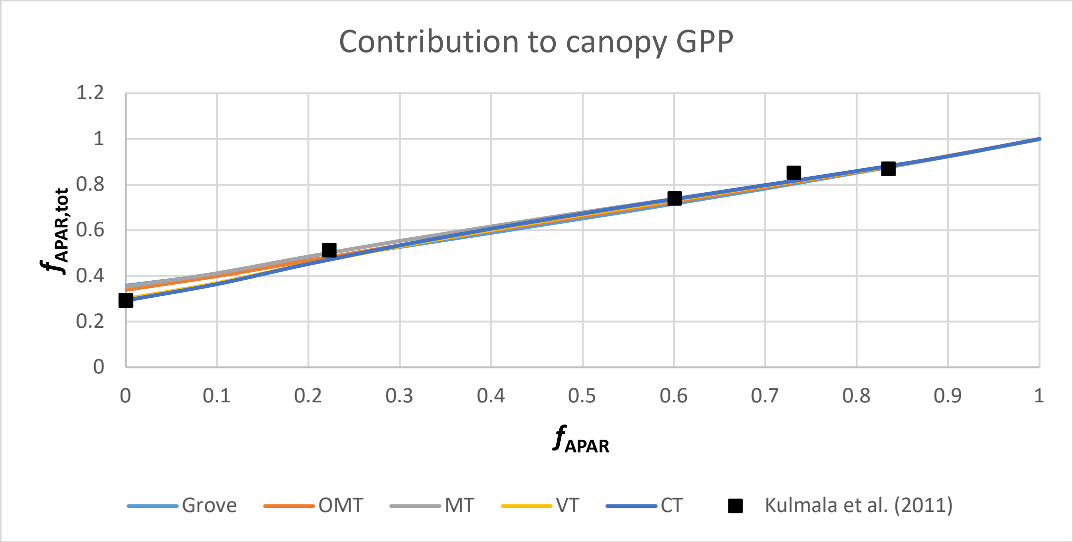

Figure S2.4. a) Comparison of ground vegetation biomass in the observations by Kulmala et al. (2011) and the current model at sub-xeric sites. b) Comparison of the relationship between tree canopy f_{APAR} and combined canopy + GV $f_{APAR,tot}$ in model and observations. The site types are OMT = grove-like, MT = herb-rich, VT = sub-xeric, CT = xeric.

Tupek et al. (2016) presented a statistical model of above- and belowground biomass of ground vegetation types, divided into grasses, herbs, shrubs, mosses and lichens. The explanatory variables were stand variables available from NFI measurements, including latitude. The key variables in all models were the main tree species (pine or spruce) and latitude. However, there was a lot of uncertainty in the prediction, the goodness of fit of the models ranging from 0.1 to 0.3.

We used open-access data from the Swedish national forest inventory (Skogsdata 2020) as input to the process-based growth model PREBAS (Minunno et al. 2019) to assess site fertility and estimate f_{APAR}. The input data required by our model was total basal area and its proportional share among species, and mean height and mean diameter per species. We used these to estimate the biomass of the three ground-vegetation species groups included in the present model. We then compared these corresponding predictions with the model by Tupek et al. (2016), which could also be evaluated for the sites.

Because of a larger number of explanatory factors, the variability in values predicted by the Tupek model was much larger than that in PREBAS combined with the current GV model. However, the order of magnitude and the overall range of variability were similar in both models (Figure S2.5).

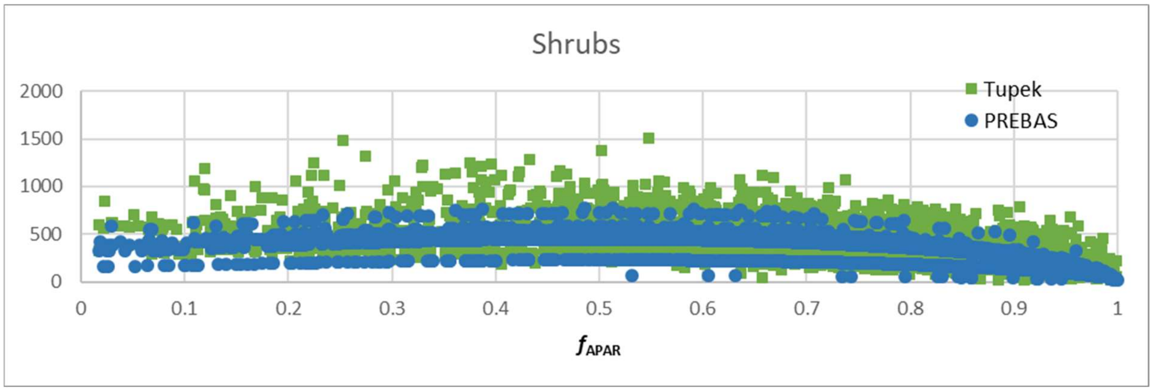

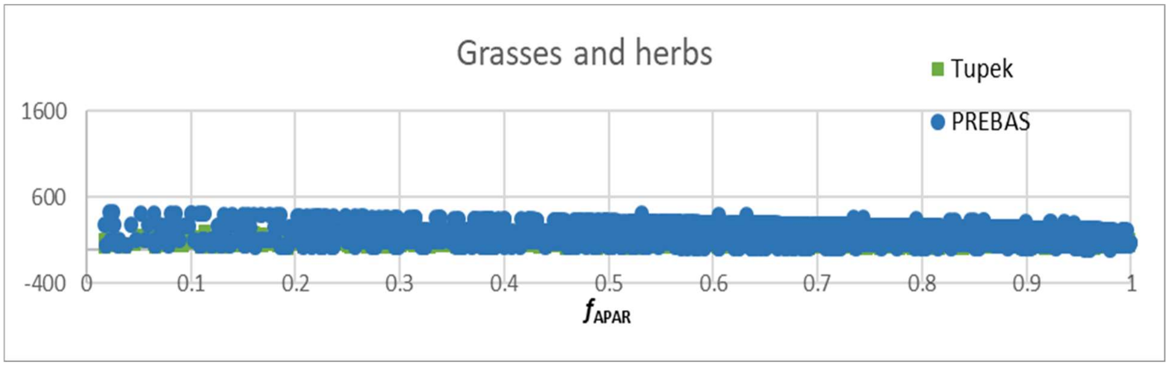

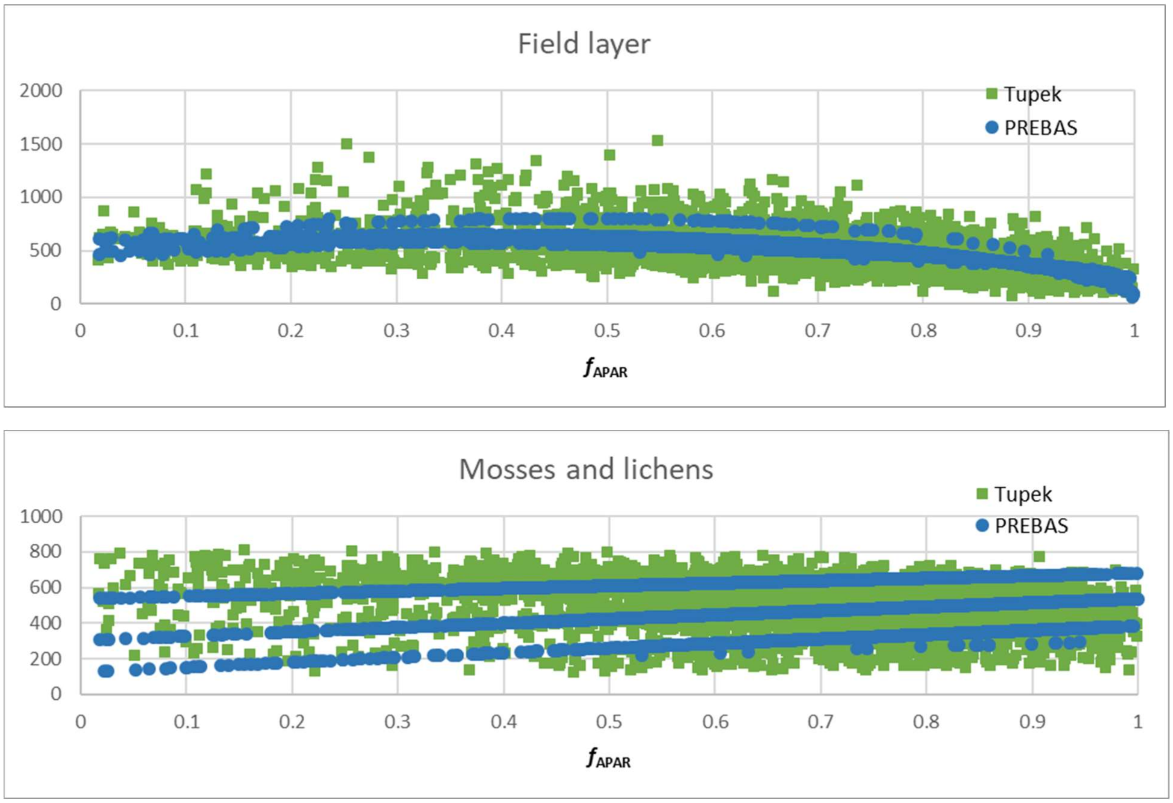

Figure S2.5. Comparison of predictions by PREBAS and the empirical model by Tupek et al. (2016) in Swedish NFI data. Field layer includes grasses, herbs and shrubs (kg C ha^{-1}), while bottom layer includes mosses and lichens.

Concluding remarks

The GV module of PREBAS is a first attempt to include GV-related carbon fluxes in the PREBAS model for regional-scale applications. Based on the large-scale summary type data used (Tonteri et al. 2005, Tähkälä 2011, Lehtonen et al. 2016), the model is largely qualitative and can be expected to provide results of appropriate order of magnitude only, rather than accurate site-specific estimates. However, the comparisons with previous empirical studies (Kulmala et al. 2011, Tupek et al. 2016) suggested that the overall order of magnitude and relations between different functional groups were consistent. Concurrently, both test data sets showed somewhat higher shrub biomass than could be predicted from our model building data, but the modelled patterns with respect to light availability agreed with data. Our model showed more variability in grasses and herbs than Tupek's model both with site type and light availability, probably reflecting the fact that these variables were not included in Tupek's explanatory factors. The understorey contribution to ecosystem GPP through light capture was remarkably similar to the results by Kulmala et al. (2011) and rather insensitive to site type, suggesting that after clear cut ground vegetation can capture 30% of the total light capture of closed stands, the ratio declining steadily as the tree canopy becomes denser. However, the results need further testing against comprehensive empirical data, such as measurements available from eddy covariance sites.

References

Kulmala L, Pumpanen J, Kolari P, Muukkonen P, Hari P, Vesala T. 2011. Photosynthetic production of ground vegetation in different-aged Scots pine (*Pinus sylvestris*) forests. Can. J. For. Res. 41: 2020–2030. doi:10.1139/X11-121.

Laine AM, Tolvanen A, Mehtätalo L, Tuittila E-S. 2016. Vegetation structure and photosynthesis respond rapidly to restoration in young coastal fens. Ecology and Evolution 6(19): 6880–6891. doi: 10.1002/ece3.2348.

Lehtonen A., Linkosalo t, Peltoniemi M, Sievänen R, Mäkipää R, Tamminen P, Salemaa M, Nieminen T, Tupek B, Heikkinen J, Komarov A 2016. Forest soil carbon stock estimates in a nationwide inventory: evaluating performance of the ROMULv and Yasso07 models in Finland. Geosci. Model Dev., 9, 4169–4183. doi:10.5194/gmd-9-4169-2016.

Minunno F, Peltoniemi M, Launiainen S, Aurela M, Mammarella I, Lindroth A, Lohela A, Minkkinen K, Mäkelä A 2016. Calibration and validation of a semi-empirical flux ecosystem model for coniferous forests in the Boreal region. *Ecological Modelling* 341:37-52.

Minunno F., Peltoniemi M., Härkönen S., Kalliokoski T., Mäkinen H., Mäkelä A. 2019. Bayesian calibration of a carbon balance model PREBAS using data from permanent growth experiments and national forest inventory. *Forest Ecology and Management* 440: 208-257. https://doi.org/10.1016/j.foreco.2019.02.041

Muukkonen P., Mäkipää R., Laiho R., Minkkinen K., Vasander H., Finér, L. 2006. Relationship between Biomass and Percentage Cover in Understorey Vegetation of Boreal Coniferous Forests. Silva Fennica 40: 231-245.

Peltoniemi, M., Pulkkinen, M., Aurela M., Pumpanen, J., Kolari, P., Mäkelä, A. 2015. A semi-empirical model of boreal forest gross primary production, evapotranspiration, and soil water – calibration and sensitivity analysis. *Boreal Environment Research* 20: 151–171.

Skogsdata 2020. Forest Statistics 2020. Official Statistics of Sweden. Swedish University of Agricultural Sciences. Umeå 2020. https://www.slu.se/en/Collaborative-Centres-and-Projects/the-swedish-national-forest-inventory/

Strengbom J, Näsholm T, Ericson L 2004. Light, not nitrogen, limits growth of the grass *Deschampsia flexuosa* in boreal forests. Can. J. Bot 82: 430-435. DOI: 10.1139/B04-017

Tonteri, T., Hotanen, J.-P., Mäkipää, R., Nousiainen, H., Reinikainen, A. & Tamminen, M. 2005. Metsäkasvit kasvupaikoillaan – kasvupaikkatyypin, kasvillisuusvyöhykkeen, puuston kehitysluokan ja puulajin yhteys kasvilajien runsaussuhteisiin. Metsäntutkimuslaitoksen tiedonantoja 946. 106 s.

Tähkälä T. 2011. Metsikön rakenteen ja hakkuiden vaikutus pintakasvillisuuteen Etelä-Suomen yleisimmillä kasvupaikkatyypeillä. Metsäekologian pro gradu-tutkielma maatalous-metsätieteiden maisterin tutkintoa varten. MSc thesis. Helsingin yliopisto. Metsätieteiden laitos.

Tupek B., Ortiz CA, Hashimoto S, Stendahl J, Dahlgren J, Karltun E, Lehtonen A. 2016. Underestimation of boreal soil carbon stocks by mathematical soil carbon models linked to soil nutrient status. Biogeosciences 13: 4439-4459. DOI: 10.5194/bg-13-4439-2016

S3. Habitat Suitability Index (HSI) of biodiversity indicators

We used six previously published habitat suitability indices (HSI) for five bird species (capercaillie (*Tetrao urogallus*), hazel grouse (*Bonasa bonasia*), three-toed woodpecker (*Picoides tridactylus*), lesser-spotted woodpecker (*Dryobates minor*), long-tailed tit (*Aegithalos caudatus*)), and one mammal species (Siberian flying squirrel (*Pteromys volans*)) (Mönkkönen et al. 2014). The HSI attained values between 0 and 1 and were calculated by inserting the stand structural variables produced by the PREBAS simulations, such as tree species absolute and relative volume, basal area, density, age, and mortality or presence of dead trees (Table S3.1), to the species-specific HSI formulas described below.

Table S3.1. Stand variable used to calculate HSI-values for each of the indicators.

**Species common name**	**Scientific name**	Total tree volume (m^3/ha)	Pine volume (m^3/ha)	Spruce volume (m^3/ha)	Deciduous volume (m^3/ha)	Density of trees (stems/ha)	Age (years)	% of volume deciduous	% of volume spruce	basal area of living trees (m^3/ha)	basal area of fresh deadwood (m^3/ha)	basal area of fresh deciduous deadwood (m^3/ha)
Capercaillie (lekking site)	*Tetrao urogallus*		x	x		x						
Hazel grouse	*Bonasa bonasia*						x	x	x			
Three-toed woodpecker	*Picoides tridactylus*	x									x	
Lesser-spotted woodpecker	*Dryobates minor*						x					x
Long-tailed tit	*Aegithalos caudatus*						x	x		x		
Siberian flying squirrel	*Pteromys volans*			x	x				x			

Here, we summarise the formulas used to calculate HSI-values for each indicator and give a short description of their interpretation, as reported by Mönkkönen et al. (2014). For further details, see Mönkkönen et al. (ibid.), Appendix 2 in the supplementary material.

Capercaillie (*Tetrao urogallus*) lekking site HSI

$HSI_{capercaillie} = w_{pine} * w_{spruce} * w_{density},$ where

Pine volume (m^3/ha)

$$w_{pine} = \begin{cases} 0, & if\ v_{pine} \le 60 \\ 0.05 * v_{pine} - 3, & if\ 60 < v_{pine} \le 80 \\ 1, & if\ v_{pine} > 80 \end{cases}$$

Spruce volume (m^3/ha)

$$w_{spruce} = \begin{cases} 0, & if v_{spruce} \le 5 \\ 0.2\ * v_{spruce} - 1, & if\ 5 < v_{spruce} \le 10 \\ 1, & if\ 10 < v_{spruce} \le 20 \\ -0.1\ * v_{spruce} + 3, & if\ 20 < v_{spruce} \le 30 \\ 0, & if v_{spruce} > 30 \end{cases}$$

Density of trees (stems/ha)

$$w_{density} = \begin{cases} 0, & if\ stems/ha\ \le 500 \\ 0.01\ * stems/ha\ - 5, & if\ 500 < stems/ha \le 600 \\ 1, & if\ 600 < stems/ha \le 800 \\ -0.005 * stems/ha + 5, & if\ 800 < stems/ha \le 1000 \\ 0, & if\ stems/ha > 1000 \end{cases}$$

The capercaillie lekking site HSI equals to one when pine volume is high (>80m^3/ha), spruce volume is at an intermediate level (10-20 m^3/ha), and stem density is intermediate (600-800 stems/ha).

Hazel grouse (*Bonasa bonasia*) HSI

$HSI_{hazel\ grouse} = w_{age} * w_{dec} * w_{spruce}$, where

Forest age (years)

$$w_{age} = \begin{cases} 0, & if\ age\ \le 20 \\ 0.1\ * age - 2, & if\ 20 < age \le 30 \\ 1, & if\ 30 < age \le 60 \\ -0.012 * age + 1.72, & if age > 60 \end{cases}$$

Proportion of deciduous trees (%) of the total tree volume

$$w_{dec} = \begin{cases} 0, & if\ p_{dec} \le 5 \\ 0.066\ * p_{dec} - 0.33, & if\ 5 < p_{dec} \le 20 \\ 1, & if\ 20 < p_{dec} \le 40 \\ -0.05 * p_{dec} + 3, & if\ 40 < p_{dec} \le 60 \\ 0, & if\ p_{dec} > 60 \end{cases}$$

Proportion of spruce (%) of the total tree volume

$$w_{spruce} = \begin{cases} 0, & if\ p_{spruce} \le 20 \\ 0.2 * p_{spruce} - 4, & if\ 20 < p_{spruce} \le 25 \\ 1, & if\ p_{spruce} > 25 \end{cases}$$

Equals to one when the age of forest is between 20 and 60 years, proportion of deciduous trees of the total tree volume is intermediate (20-40 %) and the proportion of spruce is high (>25%).

Three-toed woodpecker (*Picoides tridactylus*) HSI

$HSI_{ttwo} = w_{dw} * w_{vol},$ where

Basal area (BA; m^2/ha) of recently died trees

$$w_{dw} = 1/\left(1 + e^{-(3.55BA- \ .46)}\right)$$

Total tree volume

$$w_{vol} = \begin{cases} 0, & if\ v_{total} < 60 \\ v_{total}/200, & if\ 60 \leq v_{total} \leq 200 \\ 1, & if\ v_{total} > 200 \end{cases}$$

The value is close to one when the total volume living trees is >200 m^3/ha and basal area (m^2/ha) of fresh dead-wood is >2.5m^2/ha. The latter threshold level translates into about 20m^3/ha of deadwood in a mature stand (v_{total} > 200).

Lesser-spotted woodpecker (*Dryobates minor*) HSI

$HSI_{lswo} = w_{dw} * w_{age},$ where

Basal area (BA; m^2/ha) of recently died deciduous trees

$$w_{dw} = 1/\left(1 + e^{-(6.32BA- \ .96)}\right)$$

Forest age

$$w_{age} = \begin{cases} 0, & if\ age < 60 \\ age/200, & if\ 60 \leq age \leq 200 \\ 1, & if\ age > 200 \end{cases}$$

The value is close to one when basal area (BA; m^2/ha) of fresh deciduous dead-wood is >1.5m^2/ha and the age of the stand is >200 years

Long-tailed tit (*Aegithalos caudatus*) HSI

$HSI_{ltt} = w_{age} * w_{dec} * w_{ba},$ where

Forest age

$$w_{age} = \begin{cases} 0, & if\ age < 30 \\ 0.033 * age - 1, & if\ 30 \leq age < 60 \\ 1, & if\ age \geq 60 \end{cases}$$

Total basal area

$$w_{ba} = \begin{cases} 0, & if\ BA\ \leq 10 \\ 0.2 * BA - 2, & if\ 10 < BA \leq 15 \\ 1, & if\ BA\ > 15 \end{cases}$$

Proportion deciduous tree of total timber volume

$$w_{dec} = \begin{cases} 0, & if\ p_{dec} \leq 20 \\ 0.025\ * p_{dec} - 0.5, & if\ 20 < p_{dec} \leq 60 \\ 1, & if\ p_{dec} > 60 \end{cases}$$

Equals to one when the age of forest is >60 years, proportion of deciduous trees of the total tree volume is >60%, and total basal area of trees is >15 m^2/ha. Basal area of 15 m^2/ha translates into total volumes well above 100 m^3/ha in forests that are older than 60 years.

Siberian flying squirrel (*Pteromys volans*) HSI

$HSI_{fs} = w_{sprucevol} * w_{sprucep} * w_{dec}$, where

Spruce vol m^3/ha

$$w_{sprucevol} = \begin{cases} 0, & if\ v_{spruce} \leq 140 \\ 0.028\ * v_{spruce} - 4, & if\ 140 < v_{spruce} \leq 175 \\ 1, & if\ v_{spruce} \geq 175 \end{cases}$$

Proportion of spruce of total timber volume (%)

$$w_{sprucep} = \begin{cases} 0, & if\ p_{spruce} \leq 50 \\ 0.1 * p_{spruce} - 5, & if\ 50 < p_{spruce} \leq 60 \\ 1, & if\ p_{spruce} > 60 \end{cases}$$

Volume of deciduous trees m^3/ha

$$w_{dec} = \begin{cases} 0, & if\ v_{dec} \leq 12 \\ 0.333\ * v_{dec} - 4, & if\ 12 < v_{dec} \leq 15 \\ 1, & if\ v_{dec} > 15 \end{cases}$$

Equals to one when the volume of spruce is > 175 m^3 /ha, the proportion of spruce of the total timber volume is >60%, and the volume of deciduous trees is > 15 m^3/ha.

Refrences

Mönkkönen, M., Juutinen, A., Mazziotta, A., Miettinen, K., Podkopaev, D., Reunanen, P., Salminen, H., Tikkanen, O.-P., 2014. Spatially dynamic forest management to sustain biodiversity and economic returns. J. Environ. Manag. 134, 80–89.

S4. Age class distribution

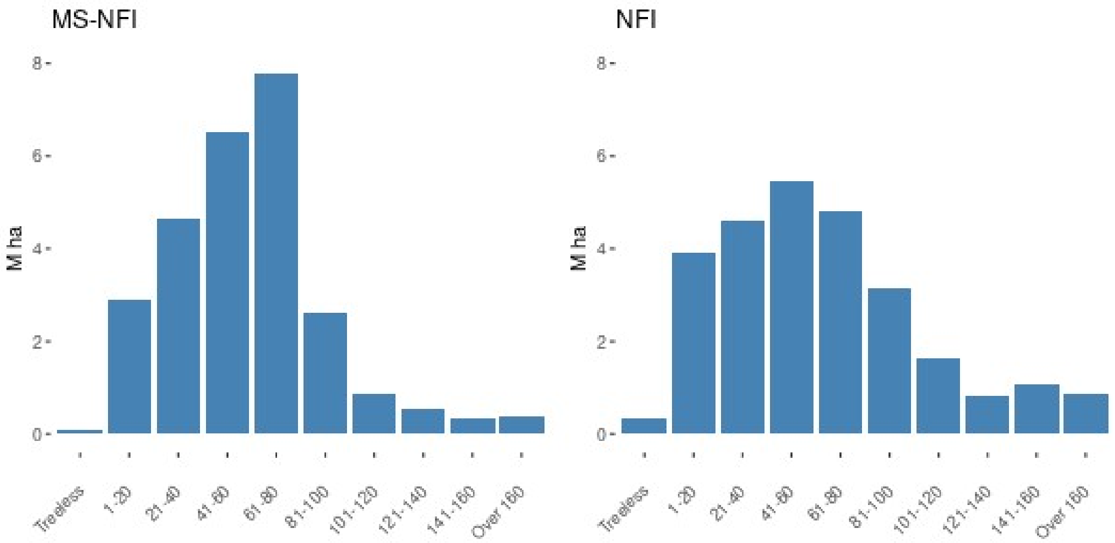

Figure S4.1. Age class distributions evaluated from multi-source NFI (left) and plot-based NFI (right). The figure shows the total area in 20-year age classes in Finland, plus the total area of treeless sites that appear between clear cut and regeneration.

S5. Stakeholder meeting

A stakeholder meeting was held by the IBC-CARBON project on 7 November 2018, where the invited 13 experts represented universities, research institutes, ministries, forest owners' associations, forest consultancy companies, companies managing nationally owned forests, and different NGOs. In addition, 9 project members and one external facilitator took part in the meeting.

The participants were sent some pre-reading material with background about the research methods and objectives of the meeting, which was expanded in introductory presentations. This included a general presentation of the research project and its objectives, and an introduction to the PREBAS model and its intended application in the project.

The main setup of the study was designed prior to the meeting, including the need to specify three alternative management strategies based on the three objectives (climate change adaptation, climate change mitigation by means of forest carbon, biodiversity conservation), and the idea of varying the realised cutting levels. The main question to the stakeholders was, what management actions they think would promote these specified objectives.

The discussion was conducted in 3 groups that were chaired by project members and included both external experts and project researchers. The groups interacted with each other during the day. When summarising the results, the researchers communicated with the experts about the possible limitations of the applied model in representing potential management actions.

The results of the meeting were summarised in a document outlining the ideas and the resulting management actions that could be used as input to the model. The document was circulated among the participants for comments.

The meeting was one in a series of stakeholder meetings organised in connection with four Finnish Strategic Research Council projects that were part of a study on knowledge and knowledge co-production (Korhonen-Kurki et al. 2022).

Refrences

Korhonen-Kurki, K., Bor, S., Faehnle, M., Kosenius, A.-K., Kuusela, S., Käyhkö, J., Pekkonen, M., Saarikoski, H., Keskinen, M. 2022. Empirical insights into knowledge-weaving processes in strategicenvironmental research. Journal of Environmental Policy and Planning 24:733-748. https://doi.org/10.1080/1523908X.2022.2044296

S6. Results

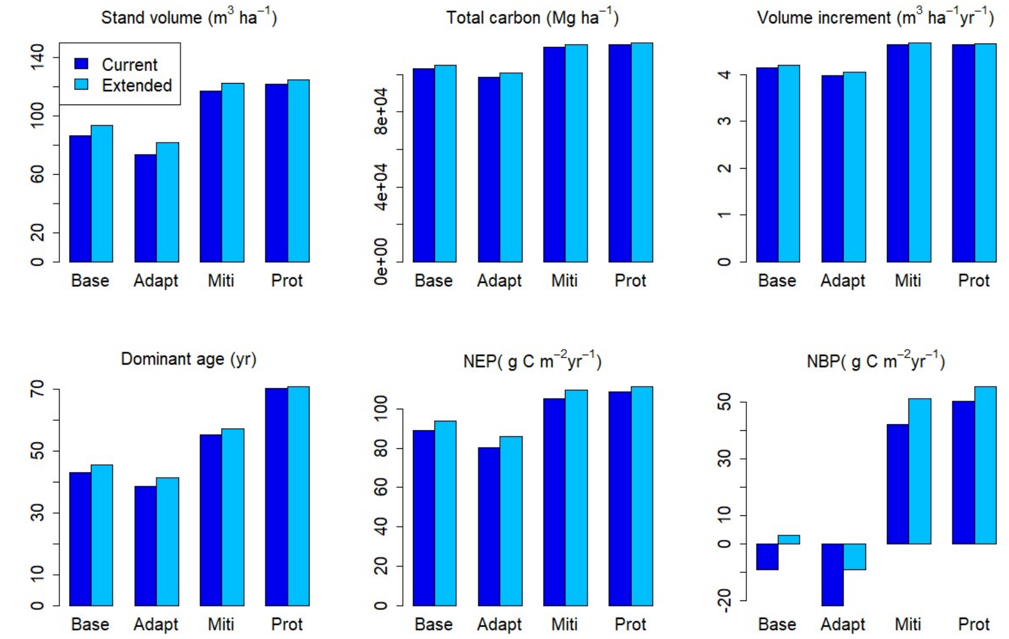

Figure S6.1a. Temporal mean values over 2021-2050 of C balance indicators under different management strategies in management-driven scenarios. Current = with current protection areas, Extended = with at least 10% set aside by all regions.

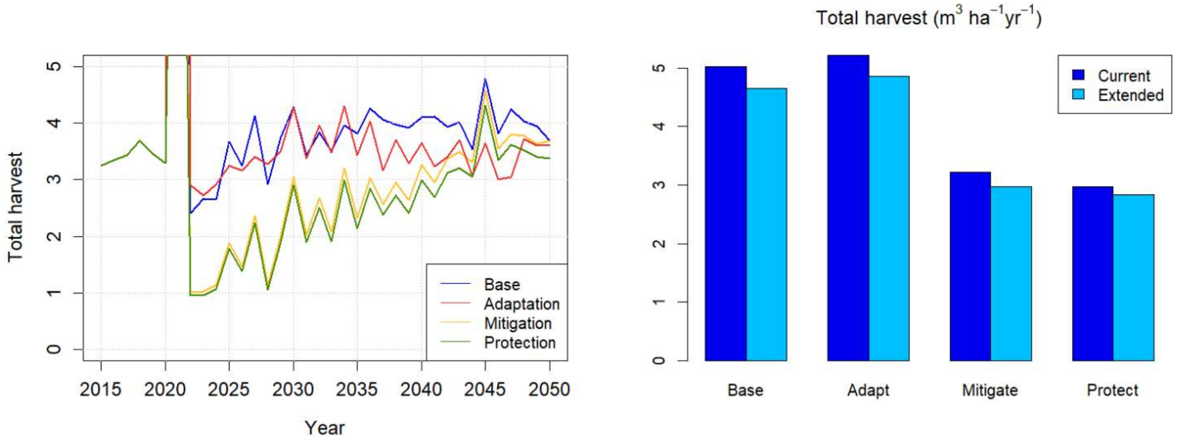

Figure S6.1.b. Left: Time development of cutting levels in management-driven scenarios from a total area of 23.4 Mha. Right: Total area-based harvest in management-driven scenarios with current and extended protected areas.

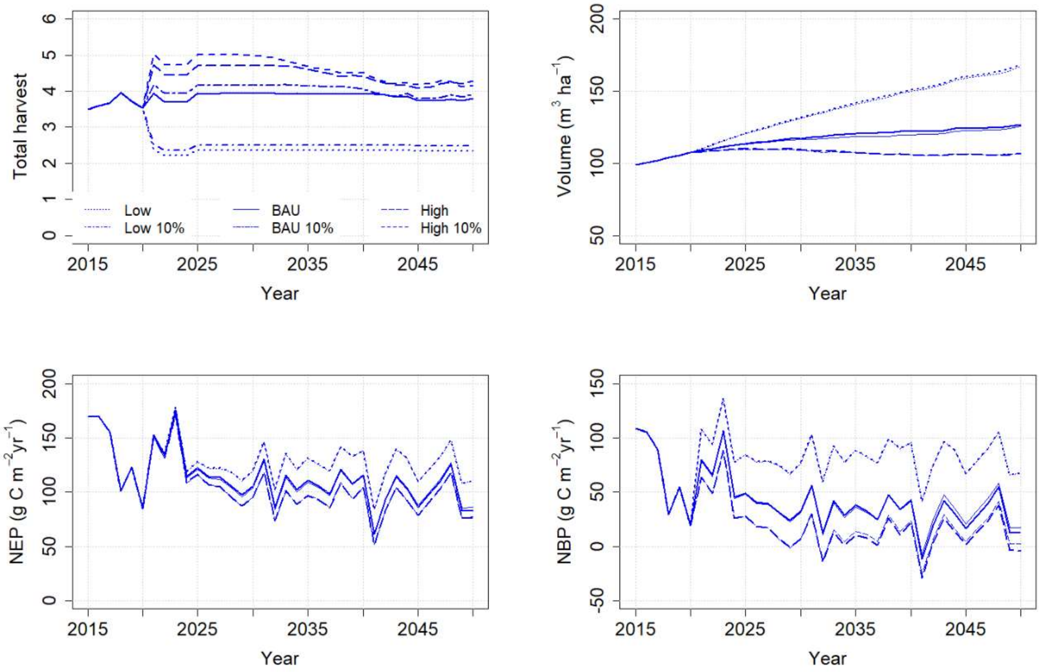

Figure S6.2 Comparison of C balance indicator variables in demand-driven scenarios under base management strategy with original and extended protected areas. Total harvests (including collected harvest residues) are shown per managed forest area (21.4 and 20.2 Mha with original and extended protected areas, respectively), other variables are shown as national averages with respect to total forest area (23.4 Mha).

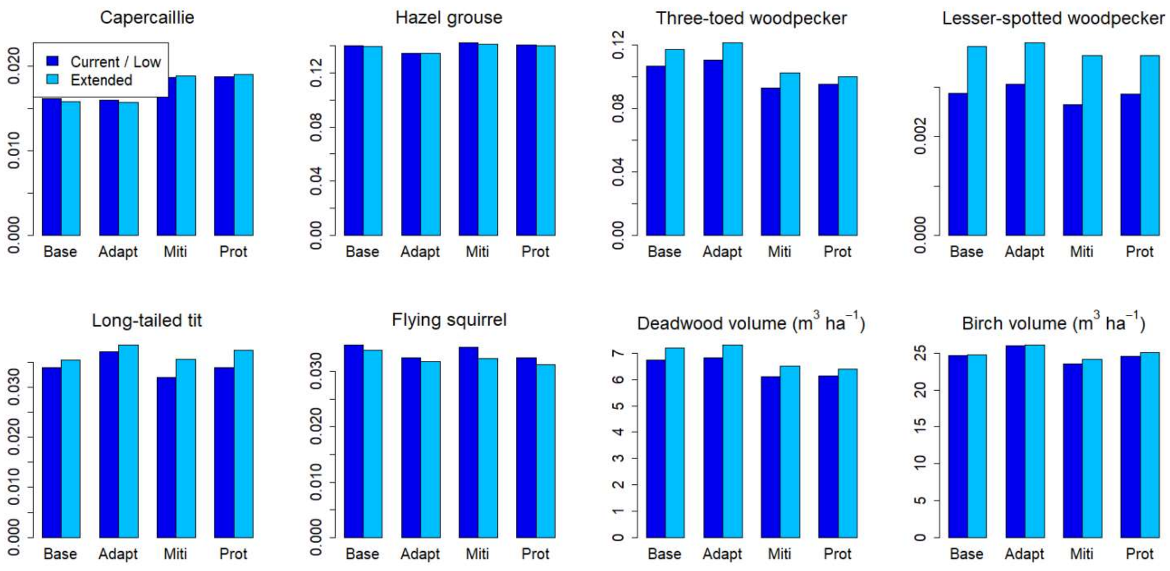

Figure S6.3 a. Biodiversity indicators with current and extended (10%) protection areas in Low harvest scenarios. Miti = mitigation scenario, Prot = protection scenario.

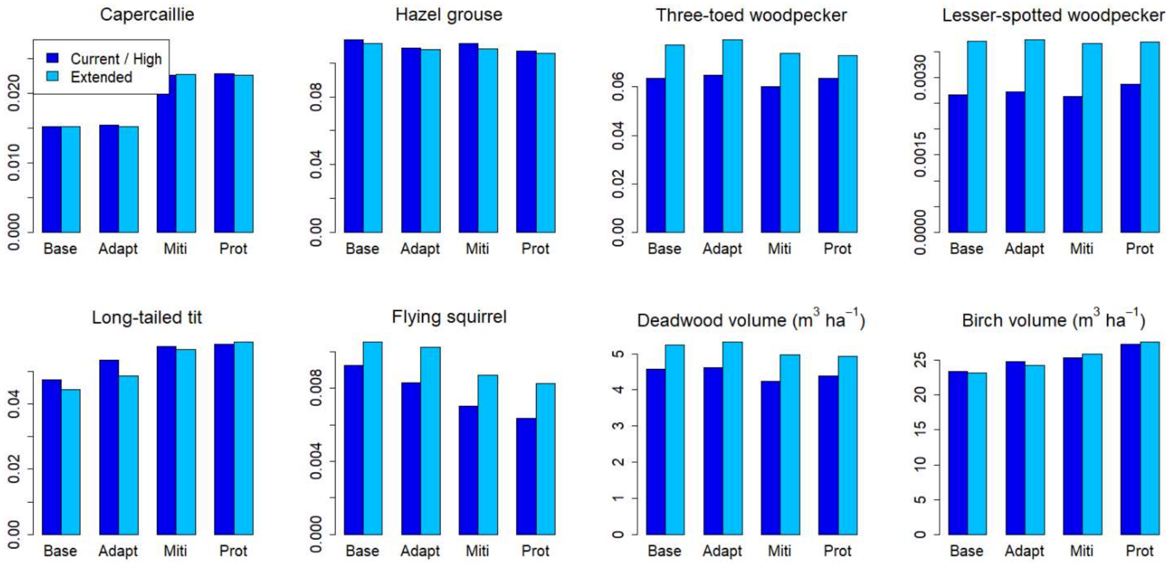

Figure S6.3 b. Biodiversity indicators with current and extended (10%) protection areas in High harvest scenarios.
